# THOUSAND-GRAIN WEIGHT 6, which is an IAA-glucose hydrolase, preferentially recognizes the structure of the indole ring

**DOI:** 10.1038/s41598-024-57506-z

**Published:** 2024-03-21

**Authors:** Tatsuki Akabane, Nobuhiro Suzuki, Kazuyoshi Ikeda, Tomoki Yonezawa, Satoru Nagatoishi, Hiroyoshi Matsumura, Takuya Yoshizawa, Wataru Tsuchiya, Satoshi Kamino, Kouhei Tsumoto, Ken Ishimaru, Etsuko Katoh, Naoki Hirotsu

**Affiliations:** 1 Graduate School of Life Sciences, Toyo University, 1-1-1 Izumino, Itakura, Oura, Gunma 374-0193 Japan; 2 Research Center for Advanced Analysis, National Agriculture and Food Research Organization, 2-1-2 Kannondai, Tsukuba, Ibaraki 305-8518 Japan; 3 Medicinal Chemistry Data Intelligence Unit, Drug Development Data Intelligence Platform Group, Medical Sciences Innovation Hub Program (MIH), RIKEN, 1-7-22 Suehiro-cho, Tsurumi-ku, Yokohama City, Kanagawa 230-0045 Japan; 4 Division of Physics for Life Functions, Faculty of Pharmacy, Keio University, 1-5-30 Shibakoen Minato-ku, Tokyo, 105-8512 Japan; 5 School of Engineering, The University of Tokyo, 7-3-1 Hongo, Bunkyo-ku, Tokyo, 113-8656 Japan; 6 Department of Biotechnology, College of Life Sciences, Ritsumeikan University, 1-1-1 Noji-Higashi, Kusatsu, Shiga 525-8577 Japan; 7 CRYO SHIP Incorporated, 1-266-3, Sakuragi-cho, Omiya-ku, Saitama, Saitama 330-0854 Japan; 8 Institute of Crop Science, National Agriculture and Food Research Organization, 2-1-2 Kannondai, Tsukuba, Ibaraki 305-8518 Japan; 9 Department of Food and Nutritional Sciences, Toyo University, 1-1-1 Izumino, Itakura, Oura, Gunma 374-0193 Japan

**Keywords:** Auxin, Plant molecular biology, Screening, X-ray crystallography

## Abstract

An indole-3-acetic acid (IAA)-glucose hydrolase, THOUSAND-GRAIN WEIGHT 6 (TGW6), negatively regulates the grain weight in rice. TGW6 has been used as a target for breeding increased rice yield. Moreover, the activity of TGW6 has been thought to involve auxin homeostasis, yet the details of this putative TGW6 activity remain unclear. Here, we show the three-dimensional structure and substrate preference of TGW6 using X-ray crystallography, thermal shift assays and fluorine nuclear magnetic resonance (^19^F NMR). The crystal structure of TGW6 was determined at 2.6 Å resolution and exhibited a six-bladed β-propeller structure. Thermal shift assays revealed that TGW6 preferably interacted with indole compounds among the tested substrates, enzyme products and their analogs. Further analysis using ^19^F NMR with 1,134 fluorinated fragments emphasized the importance of indole fragments in recognition by TGW6. Finally, docking simulation analyses of the substrate and related fragments in the presence of TGW6 supported the interaction specificity for indole compounds. Herein, we describe the structure and substrate preference of TGW6 for interacting with indole fragments during substrate recognition. Uncovering the molecular details of TGW6 activity will stimulate the use of this enzyme for increasing crop yields and contributes to functional studies of IAA glycoconjugate hydrolases in auxin homeostasis.

## Introduction

Rice (*Oryza sativa*) is one of the most important staple cereal crops worldwide and Asia’s most important crop^1^. Improving rice yield is a challenging objective that will help alleviate the ever-increasing demand for food following rapid population growth. Grain yield in rice is determined by sink size and source ability. Many genes involved in sink size such as grain number^2–4^ and grain size^5–7^ have been identified. In contrast, only a limited number of genes involved in the source capacity has been identified. For instance, the loss of *NARROW LEAF 1* (*NAL1*) increases the photosynthetic rate per leaf area^8^ and alters canopy photosynthesis^9^.

Ishimaru and Hirotsu et al. first identified *THOUSAND-GRAIN WEIGHT 6* (*TGW6*), a gene that limits the number of endosperm cells and rice grain weight^10^. Furthermore, *tgw6* from Kasalath, an indica rice landrace cultivar, has a 1-bp deletion that causes a premature stop in the gene, resulting in enhanced grain weight and yield due to loss of function. Moreover, the loss of function of TGW6 increases starch accumulation in leaf sheaths before heading, resulting in improved grain yield^10^. In addition, the *TGW6* ortholog in wheat (*Triticum aestivum*) was reported to influence grain weight^11^. Because of these agronomic benefits, *TGW6* is a predominant target for breeding and genome editing^12^.

Whereas much attention has been paid to using *TGW6* in breeding programs, the molecular basis for TGW6 activity has not been fully elucidated. In previous studies^10,13^, the structure of TGW6 was modeled based on its amino acid sequence, and putative active residues implied hydrolase activity of TGW6^10^. A docking simulation of substrate candidates was conducted using the modeled TGW6 and indicated that indole-3-acetic acid (IAA)-glucose (Glc) would be one of the substrates for TGW6. An enzyme activity assay confirmed the IAA-Glc hydrolase activity of TGW6 using recombinant TGW6^10^. To investigate further the molecular basis for TGW6 activity, we developed purification and crystallization procedures of recombinant TGW6 for structural analyses. We found that TGW6 became more stable when Ca^2+^ ions were added during the purification steps, resulting in improved quality of the TGW6 crystals^13^. The three-dimensional structure of TGW6 and substrate recognition sites needed to be investigated to ascertain the detailed molecular bases for TGW6 activity.

From another point of view, the hydrolysis of IAA glycoconjugates by TGW6 has been considered to function in auxin metabolism. Auxin is synthesized, stored and inactivated by a multitude of parallel pathways that are all tightly regulated. Auxin conjugates are thought to play essential roles as storage forms for the active plant hormone IAA. Surprisingly, the free form of IAA is only up to 25% of the total amount of IAA in plant tissue. Most IAA is present as a storage form conjugated via ester or amide bonds with sugars and amino acids or peptides, respectively^14^. Many functional studies have concluded that auxin conjugation functions during plant development and/or in response to the environment through auxin homeostasis^15,16^. Further, the molecular mechanism of the auxin amide conjugate hydrolases was well studied, such as IAA- leucine resistantlike gene 2 (ILL2) that is known as an IAA-amino acid hydrolase^17^. The study described leucine (Leu)-175 determines the substrate specificity, and the catalytic dimetal center and the conserved glutamate (Glu)-172 contribute the substrate recognition and enzyme activity^17^. However, the mechanism for hydrolysis of auxin glycoconjugates has remained largely unknown. Providing details about the molecular mechanism for TGW6 activity will contribute not only to more extensive agricultural applications of the enzyme but also toward a basic understanding of the role of this class of enzymes for auxin homeostasis.

We, therefore, decided to gather fundamental information about TGW6 by determining its three-dimensional structure and investigating the interaction between ligands and the enzyme. We used X-ray crystallography to determine the protein’s three-dimensional structure since several protein crystal structures indicating high sequence identity with TGW6 have been solved by this method^10,18,19^, and we hypothesized that the structure of TGW6 could be determined by molecular replacement. Enzyme-ligand interactions were investigated by measuring the protein’s thermostability in the presence or absence of ligands using thermal shift assays. Additionally, fluorine nuclear magnetic resonance (^19^F NMR) fragment screening, an effective method frequently used in pharmaceutical drug discovery^20^, was used to investigate compounds interacting with TGW6 since the technique efficiently evaluates many compounds that might bind to TGW6. Further, interacting hit compounds were subjected to in silico analysis to study the character of the compounds and their interaction with TGW6. In this study, we show the importance of the IAA substructure for substrate interaction with TGW6. Our findings provide new insight into the enzyme activity mechanism of TGW6, additional applications for TGW6 and the role of auxin glycoconjugate hydrolysis in plant growth and development.

## Results

### The structural analysis of TGW6

**Figure 1:**
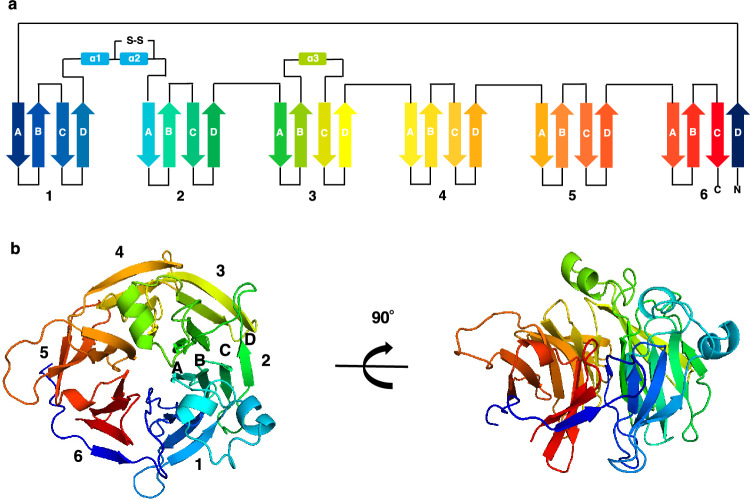
Overview of TGW6 structure. (**a**) Topology models of TGW6 structure (PDB: 8KG3). (**b**) A schematic model of the crystal structure of TGW6 (PDB: 8KG3). The numbers 1–6 indicate the blade structure number. The letters A to D show the location of each β-strand within a blade structure.

Based on our previous report, we used procedures for the large-scale purification and crystallization of recombinant TGW6^13^. The obtained crystals were used for the X-ray diffraction experiment in which X-ray diffraction data were acquired with a resolution of 2.6 Å (Table 1). We determined the crystal structures of the recombinant TGW6 (Protein Data Bank (PDB): 8KG3) in the space group of *P*1, and the asymmetric unit of the crystal contained 12 TGW6 molecules based on a *V*_M_ value of 3.26 Å^3^/Da (Table 1 and Fig. 1)^13^. The Root Mean Square Deviation (RMSD) of the corresponding atomic positions in 12 molecules were calculated less than 0.5 Å by the DALI server^21^. Since no significant difference in the overall structure among 12 molecules was observed, molecule A was used for the subsequent analysis discussed below.

**Table 1:** Crystallographic data collection and refinement statistics of TGW6.

Spring-8 Beam line	BL26B1
Diffraction data	
Wavelength (Å)	0.999994
Temperature	100 K
Crystal-detector distance (mm)	180
Rotation range per image	0.1°
Exposure time per image (sec)	0.3
Space group	*P*1
Cell parameters	a = 105.982 Å, b = 108.520 Å, c = 123.071 Å α = 94.143°, β = 98.736°, γ = 97.232°
Resolution range (Å)	49.72–2.60 (2.64–2.60)
No. of reflections	586,350 (30,303)
No. of unique reflections	161,474 (8,003)
Completeness (%)	98.3 (98.1)
*R*_*merge*_ (%)	9.6 (73.6)
Redundancy	3.6 (3.8)
*I*/σ (*I*)	8.4 (1.4)
CC (1/2)	99.3 (58.1)
Refinement and structure model	
*R*_*work*_/*R*_*free*_ factor	0.225/0.260
No. of molecules	12
Average B-factor (Å^2^)	52.17
RMSD	
Bond length (Å)	0.0132
Bond angles (°)	1.66
Ramachandran plot (%)	
Favored	94.90
Allowed	4.34
Outliers	0.76

Values in parentheses are for the highest-resolution shell.

**Figure 2:**
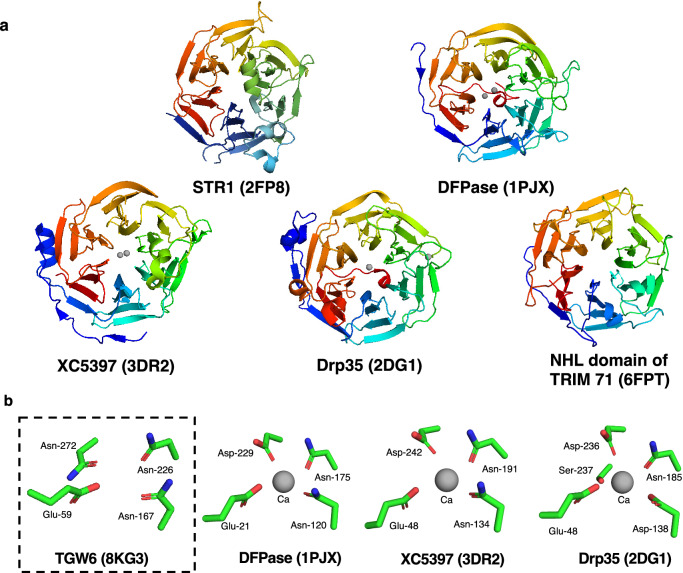
Structural comparison of TGW6 and structurally similar proteins. (**a**) Schematic models of proteins structurally similar to TGW6 (PDB: 8KG3). STR1; strictosidine synthase (PDB: 2FP8), DFPase; diisopropylfluorophosphatase (PDB: 1PJX), XC5397; gluconolactonase (PDB: 3DR2), Drp35; drug resistance protein 35 (PDB: 2DG1), NHL domain of TRIM71; the NHL domain of E3 ubiquitin-protein ligase tripartite motif-containing protein 71 (PDB: 6FPT). (**b**) Conserved putative Ca^2+^-binding sites in TGW6 and similar proteins. The gray spheres indicate the locations of Ca^2+^. The side chains of conserved Ca^2+^-binding residues are shown as stick models with colors denoting oxygen (red), carbon (green), and nitrogen (blue).

Using the DALI server, we conducted a structural similarity search for TGW6^21^. The ten best proteins showing similarity to the structure of TGW6 based on their Z-scores were tabulated (Supplementary Table S1 and Fig. 2a). According to the result of structural similarity search, the structure of TGW6 was classified into six-bladed β-propeller structure. Furthermore, some proteins with structural similarity to TGW6, such as diisopropylfluorophosphatase (DFPase)^22^, gluconolactonase (XC5397)^23^ and drug resistance protein 35 (Drp35)^24^, retain calcium (Ca^2+^) (Fig. 2b). The Ca^2+^-chelating residues were partially conserved in TGW6 as the other proteins (Glu-59, asparagine (Asn)-167, Asn-226 and Asn-272) (Fig. 2b and Supplementary Fig. S1); however, the acidic residue in the Ca^2+^-binding site of TGW6 had only Glu-59, whereas at least two acidic residues form the Ca^2+^-binding site in the other Ca^2+^-binding protein^22,25–27^. To confirm the presence of Ca^2+^ at the site of TGW6, we obtained anomalous diffraction data at 3.06801 Å, a peak wavelength for calcium atoms, but we could not find an obvious peak in the anomalous map even though the sulfur ion peak that also causes anomalous diffraction by irradiating relatively near an X-ray wavelength (~ 5.0 Å)^28^ was confirmed for the side chain of the sulfur-containing amino acid (Supplementary Table S2 and Fig. S2). Our results showed that a Ca^2+^ ion was not present at the putative Ca^2+^-binding site of TGW6 (Fig. 2b and Supplementary Fig. S2), and thus, our final model lacks Ca^2+^ ions. In summary, we confirmed that TGW6 had significant structural similarity to many proteins classifying into six bladed β-propeller, but the structural difference including the lack of a Ca^2+^-binding site in TGW6 was also found.

### Interaction analyses of substrates and fragment compounds in the presence of TGW6

**Figure 3:**
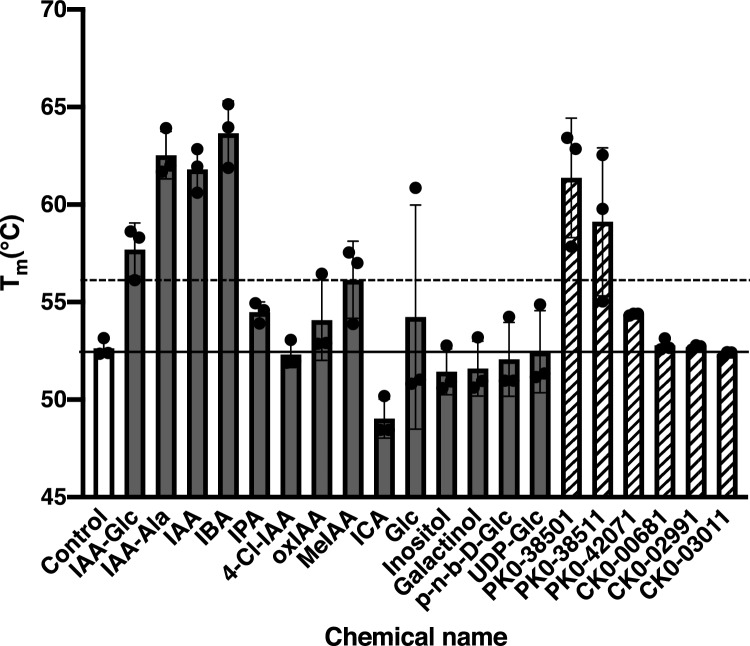
Thermal stability of TGW6 with various small compounds. The white bar is the T_m_ point of TGW6 in the control condition (no ligand). Black bars are the T_m_ points of TGW6 in the presence of a substrate, enzyme products or their analogs. The gray and hatched bars are T_m_ points of TGW6 in the presence of the six hit fragments. The horizontal solid line is the T_m_ point in the control condition, and that of dashed line is the T_m_ shifted temperature. This figure shows the standard deviations of T_m_ points for three independent experiments as error bars, and the plot indicates the T_m_ value for each experiment. For the measurements, the final concentration of recombinant TGW6 was adjusted to 27.6 μM with a 55.0 μM ligand solution.

To investigate the substrate preference of TGW6, we analyzed the interaction of TGW6 with several classes of ligands: (an authentic substrate (IAA-Glc), enzyme products (IAA and Glc) and their analogs (IAA-alanine (IAA-Ala), indole 3-butyric acid (IBA), methyl indole 3-acetate (MeIAA), indole-3-pyruvic acid (IPA), oxindole 3-acetic acids (oxIAA), 4-chloroindole 3-acetic acid (4-Cl-IAA), indole 3-carboxylic acid (ICA), inositol, uridine diphosphate glucose (UDP-Glc), p-nitrophenyl-β-D-glucopyranoside (p-n-b-D-Glc) and galactinol)) using a thermal shift assay in the UNCLE platform (Unchained Labs, Pleasanton, CA). Thermal shift assays evaluate the ligand-enzyme interaction by monitoring alterations in protein thermostability with or without ligands^29^. The protein’s melting temperature (T_m_) was defined as the mid-temperature point of the minimum and maximum limits of a barycentric mean (BCM) fluorescence curve and used as a benchmark of protein thermostability (Supplementary Fig. S3). Each T_m_ value was compared between the control condition (no ligands) and the samples (mixture with a ligand). When the T_m_ point increased by more than 3 °C by adding the ligand, we defined it as the T_m_ value shifted by increasing of thermostability of TGW6. As a result, two IAA conjugates, IAA-Glc and IAA-Ala, displayed T_m_ shifts at approximately 5 and 10 °C, respectively. The enzyme product IAA and some of its analogs, IBA and MeIAA, also increased the T_m_ point by more than 3 °C (Fig. 3). Whereas some of the ligand compounds containing an indole ring had a T_m_ shift, Glc or its analogs and Glc conjugates did not influence the thermostability of TGW6 (Fig. 3). These results suggested that TGW6 tends to bind to an IAA substructure rather than a Glc moiety and is capable of interacting with other IAA conjugates as well.

On the other hand, drug discovery methods are useful for comprehensively evaluating ligand-enzyme interactions since they use chemical libraries to confirm the binding of many compounds against a target protein. Especially, drug discovery using ^19^F NMR fragment screening is one of the most efficient approaches among several drug discovery strategies^30,31^. Thus, we explored the fragment compounds that interact with TGW6 using fluorinated fragment libraries (Kishida Chemical, Osaka, Japan). The NMR signal intensity of 1,134 fluorine fragment compounds in the chemical libraries was measured as described in the Methods section. The signal intensity of the fragment compounds is markedly reduced when the fragments bind to proteins (Supplementary Fig. S4). As a result of ^19^F NMR fragment screening, the signal intensities of six compounds were considerably reduced (> 45%) in the presence of TGW6. These six fragment compounds were defined as the “hit fragments” of ^19^F NMR fragment screening (Table 2). Hit fragments were validated using a fingerprint: Pharmacophore Fingerprint (PHFP), which represents differences in the type of interaction with proteins. The cluster number for the fingerprint is shown in Table 2, and the same number represents fragments having similar characteristics in the fingerprint. The results indicated that the three hit fragments with an indole ring, PK0-38511, PK0-38501 and PK0-42071, were all classified into cluster 264 based on the PHFP (PHFP 264). In addition, the other fragments in the PHFP 264 cluster showed relatively higher signal decay than the other clusters (Supplementary Table S3). We performed a competition assay based on ^19^F NMR measurements to validate the thermal shift assay results, testing two hit fragments and IAA-Glc in the presence of TGW6 (Supplementary Table S4). We selected PK0-38511 as a representative hit fragment that contained an indole ring and CK0-00681 as a candidate without an indole ring based on NMR signal decay data (Table 2). As a result, the signal decrease ratio of PK0-38511 was recovered at about 30% by adding IAA-Glc, but that of CK0-00681 was not influenced. This result implied that PK0-38511 was bound to the same site as IAA-Glc, thereby competing with the substrate, but CK0-00681 interacted with a different site because it did not compete with the substrate (Supplementary Table S4). This result also emphasized the importance of the indole moiety for recognition by TGW6. These results supported that TGW6 preferentially interacted with indole fragments.

**Table 2:** Small molecule compounds that bind to TGW6.

2D structure	ID	Chemical name	ΔIntensity (%)	Cluster no. PHFP
	CK0-00681	2-[(2-Fluoro-4-nitrophenoxy)methyl]tetrahydrofuran	− 100.0	47
	CK0-02991	1-(3-Fluoro-2-nitrophenyl)piperidin-4-ol	− 57.9	29
	PK0-38511	2-(7-Fluoro-1H-indol-3-yl)acetic acid	− 50.3	264
	PK0-38501	2-(4-Fluoro-1H-indol-3-yl)acetic acid	− 46.5	264
	PK0-42071	3,3,3-Trifluoro-2-(5-fluoro-1H-indol-3-yl)propanoic acid	− 46.0	264
	CK0-03011	4-(2-Fluoro-4-nitrophenoxy)tetrahydro-2H-pyran	− 45.4	48

ID indicates the ID number of the fluorine fragment in the chemical library (Kishida Chemical).

Δ Intensity is the reduced percentage of the NMR signal intensity in the ^19^F NMR fragment screening.

Cluster No. PHFP is the cluster number based on the pharmacophore fingerprint.

The final concentration of recombinant TGW6 was adjusted to 10.0 μM, and the CF-, CF_2_- and CF_3_-containing molecules were diluted to 50, 25 and 17.5 μM, respectively, for the measurements.

We also analyzed the interaction of hit fragments with TGW6 based on thermal shift assays (Fig. 3 and Supplementary Fig. S3). Among the hit fragments, only fragments that were classified into the PHFP 264 group showed a tendency for higher T_m_ temperatures (Table 2 and Fig. 3). In particular, PK0-38511 and PK0-38501, indole acetic acid relatives, had T_m_ shifts of approximately 9 and 7 °C, respectively. Finally, our results emphasized that TGW6 preferably recognized the indole fragments among the variety of fragments represented in this chemical library based on ^19^F NMR fragment screening and thermal shift assays.

### Docking simulation of ligands to TGW6

**Figure 4:**
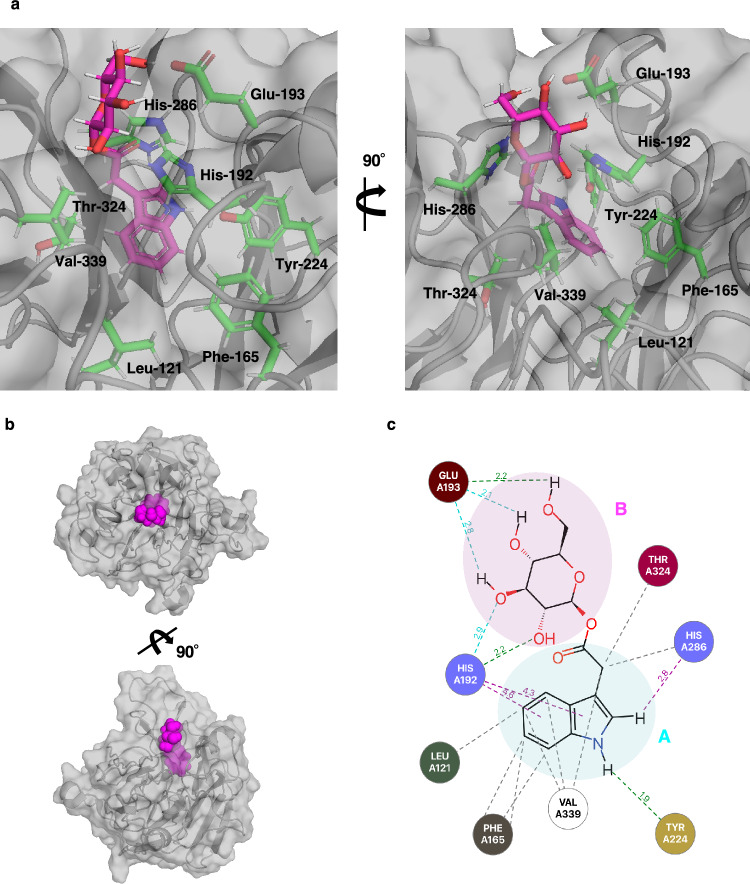
Docking pose of IAA-Glc in the presence of TGW6. (**a**) A portion of TGW6 is represented as a drawing and the surface model is shown in gray. IAA-Glc is shown as a stick model with atom colors denoting oxygen (red), carbon (purple), nitrogen (blue) and hydrogen (gray). The side chains of key residues interacting with IAA-Glc are shown as stick models with colors denoting oxygen (red), carbon (green), nitrogen (blue) and hydrogen (gray). (**b**) The binding position of IAA-Glc in the TGW6 crystal structure (PDB: 8KG3). TGW6 is schematically shown as a gray surface model. The simulated binding of IAA-Glc is represented as magenta spherical models. (**c**) The interaction map of IAA-Glc in the presence of TGW6. Interacting residues are represented as colored circles. The dotted lines indicate the kind of interaction and the position of interaction: hydrophobic interaction (gray), aromatic-aromatic contacts (purple), hydrogen bonds and weak hydrogen bonds (green and light blue), respectively. The colored circle in cyan indicates subpocket A and that in magenta denotes subpocket B.

To support the hypothesis that TGW6 preferably recognizes indole fragments and is capable of interacting with the other auxin conjugates as well as IAA-Glc, we conducted a molecular docking simulation using our TGW6 crystal structure and ligand compounds that interacted with TGW6 in the thermal shift assays and the ^19^F NMR experiment. Firstly, the docking pose of IAA-Glc was simulated when the IAA portion of the chemical structure bound to the inside of the molecule around phenylalanine (Phe)-165, histidine (His)-192 and tyrosine (Tyr)-224 in TGW6 (subpocket A; the interior portion of the nipped pocket), whereas that of Glc interacted at the site relatively close to the molecular surface around Glu-193, arginine (Arg)-287 and Glu-343 (subpocket B; the outer portion of the nipped pocket) (Fig. 4). Notably, the IAA moiety bound to fill the shape of subpocket A, whereas subpocket B still had a vacant space (Supplementary Fig. S5 a). The electrostatic complementarity showing the degree of electrostatic match to the protein^32^ showed that the interaction of the IAA moiety against TGW6 was more suitable compared to another ring structure (*e.g.*, the nitrophenyl group of p-n-b-D-Glc) (Supplementary Fig. S5b and c). These results supported our hypothesis that TGW6 possibly interacts with not only IAA-Glc but also the other auxin conjugates, such as auxin ester conjugates; IAA-*myo*-inositol^33^, IBA-Glc^34^ or IAA amide conjugates; IAA-aspartate (IAA-Asp) and others^35^, even if the indole substructure is necessary for ligand recognition.

The nonenzymatic isomerization of IAA-Glc occurs (1-*O*-indole-3-ylacetyl-β-D-glucose; 1-*O*-IAA-Glc) in other IAA-Glc structural isomers (2-*O*-indole-3-ylacetyl-β-D-glucose; 2-*O*-IAA-Glc, 4-*O*-indole-3-ylacetyl-β-D-glucose; 4-*O*-IAA-Glc and 6-*O*-indole-3-ylacetyl-β-D-glucose; 6-*O*-IAA-Glc)^36^. We conducted a docking simulation of 2-*O*-IAA-Glc, 4-*O*-IAA-Glc and 6-*O*-IAA-Glc in the presence of TGW6. We found that those structural isomers are also likely to bind to the pocket in TGW6 in the same manner as 1-*O*-IAA-Glc (Supplementary Fig. S6). Therefore, TGW6 may be able to recognize several substrate isomers. Notably, the predicted docking mode of the three structural isomers of IAA-Glc also showed that the IAA moiety interacted with subpocket A and that of Glc bound to subpocket B. These results supported our hypotheses that (1) TGW6 possibly has a broad substrate preference and (2) the protein preferentially recognizes the indole substructure during substrate binding despite changing the connection with the Glc moiety.

The interaction map of IAA-Glc in the presence of TGW6 suggested that Tyr-224 was involved with hydrogen bonds, His-192 and His-286 interacted as aromatic-aromatic contacts, and Leu-121, Phe-165, His-286, threonine (Thr)-324 and valine (Val)-339 were engaged in hydrophobic interaction with the IAA substructure (Fig. 4c). The map also predicted that His-192 and Glu-193 hydrogen bonded with the Glc moiety (Fig. 4c and Table 3). To validate the importance of the key residues interacting with the docked IAA-Glc, a molecular dynamics simulation (MD simulation) was performed using the Flare software (Supplementary Table S5 and Supplementary Fig. S7). This analysis identified the atom involved in the contact, the type of interaction and the ratio of the period during which the interaction existed per simulation time. These results showed that the MD simulation identified the most important residues in the docking simulation, supporting the consequence of the docking simulation (Table 3).

**Table 3:** The interacting residues by docking simulation.

2D structure	ID/Name	Interacting residues
	IAA-Glc	Leu-121, Phe-165, ***His-192***, **Glu-193**, **Tyr-224**, ***His-286***, Thr-324, Val-339
	IAA-Ala	***His-192***, ***His-286***, Arg-287, Val-339
	IAA	Leu-121, Phe-165, ***His-192***, **Tyr-224**, Thr-242, ***His-286***, Val-339
	IBA	Leu-121, Phe-165, Arg-189, ***His-192***, **Thr-196**, Thr-242, ***His-286***, Val-339
	MeIAA	Phe-165, Arg-189, ***His-192***, **Tyr-224**, Thr-242, ***His-286***, Val-339
	PK0-38511	Leu-121, Phe-165, Arg-189, ***His-192***, **Tyr-224**, Thr-242, ***His-286***
	PK0-38501	Leu-121, Phe-165, Arg-189, ***His-192***, **Tyr-224**, Thr-242, ***His-286***, Val-339

ID/Name is the ID number of the fluorine fragment compound in the chemical library or the name of the compounds.

Interacting residues shown as underlined letters identify residues involved in hydrophobic interactions with the ligand. Residues shown as bold font identify residues involved in hydrogen bonding and aromatic-aromatic contacts with the ligand. The residues shown in italics indicate the common residues for interaction with all ligands.

In addition, many putative interacting residues were common among all selected indole compounds (Table 3). The predicted interaction of TGW6 and the IAA moiety in this study was similar to other proteins recognizing IAA in previous reports (Table 3, Fig. 4 and Supplementary Fig. S8)^33,37^. This information supported the reliability of the simulation results. In summary, the binding pose of IAA-Glc was predicted such that the IAA moiety would bind to subpocket A and supported the crucial nature of the indole substructure for recognition by TGW6 in this region. Further, our results also supported the possibility that the other IAA conjugates, such as IAA-Ala, can bind to the active pocket in TGW6 based on the docking pose and the shape of the pocket.

## Discussion

In this study, we used X-ray crystallography, thermal shift assays and ^19^F NMR fragment screening to analyze the molecular basis for TGW6 activity. Potential substrates and hit fragments were subjected to in silico study to determine the characteristics of the selected compounds and to predict their docking pose with TGW6. The crystal structure of TGW6 was determined at 2.6 Å resolution and exhibited a six-bladed β-propeller structure. Based on the thermal shift assays and ^19^F NMR fragment screening, TGW6 was shown to interact preferably with indole compounds during substrate recognition. The docking simulation showed the putative binding modes for the substrate and selected fragments with TGW6. These results supported the importance of the IAA substructure for interaction with TGW6.

According to the result of structural analysis, the overall structure of TGW6 was similar with many proteins that were classified into six-bladed β-propeller structure. In contrast, the highly conserved Ca^2+^-binding motif in structurally similar proteins was not completely conserved in TGW6, and a Ca atom was not found at the site using single-wavelength anomalous diffraction. Therefore, these results implied that the Ca^2+^ ion does not bind at the partially conserved Ca^2+^-binding site in TGW6 or that the binding affinity was very weak even if the ion interacted. In addition, we found differences in the protein structure of TGW6 and the predicted docking mode of IAA-Glc between our study and a previous study^10^. In the previous study, the modeled structure of TGW6 was built based on the protein’s amino acid sequence, and the docking pose of IAA-Glc was simulated using computational chemistry software^10^. When we compared the protein structure of TGW6 and the docking pose of IAA-Glc against the protein based on the two studies, the shape of the active site varied, whereas the overall structure was similar. Further, the predicted docking direction of IAA-Glc seemed to be flipped (Supplementary Fig. S9). In our study, the structure of TGW6 was determined by X-ray crystallography, and the docking simulation was conducted using the analyzed crystal structure and the latest computational chemistry software. As a result, we propose that our study provides new and more precise insight into TGW6 structure and its mechanism of action.

^19^F NMR fragment screening is one of the most efficient drug discovery strategies^30,31^. We used the ^19^F NMR in this study to explore many possible interacting compounds. Our experiments identified six hit fragments from 1,134 test compounds. Three candidates had an indole ring and were classified into the same pharmacophore cluster (PHFP264). The chemical library contained 68 fragments with indole rings since the percentage of indole fragments in the library was around 6%. Our results indicated that the hit fragments for TGW6 were properly selected from the fragments library even though indole fragments were hit with high reproducibility. This approach, however, is not sufficiently versatile to evaluate the substrate preference for all enzymes yet, because the result would generally depend on the kind of enzyme activity and/or content of the chemical library. Thus far, using several types of chemical libraries or designing an original chemical library based on the characteristics of each target enzyme will promote our understanding of which fragment compounds interact with the target protein. Hence, ^19^F NMR fragment screening is an effective approach, not only for drug discovery but also for seeking unknown protein substrates and/or whose functions have not been revealed yet.

From the ^19^F NMR experimental results, the thermal shift assay and the docking simulation consistently supported the importance of an indole ring for substrate recognition by TGW6. In contrast, the hit fragments used in the ^19^F NMR experiment without an indole ring did not interact in the thermal shift assay, although they indicated NMR signal decay in the NMR assay. In the NMR experiment, ligand–protein interaction was evaluated based on the NMR signal from the fluorine atom in the fragment, yet interaction was assessed by the thermostability of the protein in the thermal shift assay. Fragments tended to show non-specific binding due to the small molecular weight so that hit fragments in the NMR assay contained several sorts of compounds with varied pharmacophore characteristics (Table 2). In contrast, protein thermostability is not always influenced by contacts with ligands but also depends on changes in the binding energy due to the interaction^38^. Thus, only two indole fragments were narrowed down from the six fragments tested by the thermal shift assay.

Results from the thermal shift assays and the ^19^F NMR fragment screening showed that TGW6 preferably interacted with indole compounds, whereas TGW6 did not effectively contact Glc or Glc conjugates. These findings demonstrating the importance of the indole substructure in the interaction with TGW6 were structurally and/or electrostatically supported by computational docking analysis based on the binding pose of selected indole compounds with TGW6 (Fig. 4, Table 3, Supplementary Fig. S5 and S8). In addition, IAA-Ala, IBA and MeIAA also showed interaction with TGW6 in thermal shift assays (Fig. 3), and the shape of the pocket was predicted to have a vacant space (Supplementary Fig. S5). These results suggested that TGW6 possibly interacts with other auxin conjugates except IAA-Glc, such as auxin ester conjugates; IAA-*myo*-inositol^33^, IBA-Glc^34^ or IAA amide conjugates; IAA-aspartate (IAA-Asp) and others^35^, even if the indole substructure is necessary for ligand recognition.

A broad substrate specificity has been reported for enzymes involving auxin conjugates; OsIAGT1, an auxin glycosyltransferase producing auxin-glucose conjugates in rice^34^, TaIAR3, an auxin amide-conjugate hydrolase in wheat (*Triticum aestivum*)^35^ and GH3, an acyl acid synthase producing auxin amide-conjugates in several plant species^39–41^. In these reports, the target enzymes were expressed heterologously, purified and the enzyme activity was assessed using several substrate analogs such as IAA, IBA, IPA, ICA, naphthalene-1-acetic acid (NAA), 2,4-dichlorophenoxyacetic acid (2,4-D) and their amide or ester conjugates. The results showed that the enzymes were active with several IAA analogs, synthetic auxin and/or various auxin conjugates (auxin-amino acid conjugates as well as auxin-glycoconjugates)^34,35,39–41^. Conjugation of auxin and hydrolysis of auxin conjugates have been proposed to maintain the cellular auxin level for proper development and/or in response to the surrounding environment^16^. In practice, a family of auxin amide-conjugate hydrolases has been shown to play an important role in mediating flower pedicel abscission in tomatoes^42^. A triple mutant of a family of auxin amide-conjugate hydrolases, ILR1, IAR3 and ILL2, had reduced sensitivity for exogenous IAA, shorter hypocotyls and fewer lateral roots than wild type on an un-supplemented medium^43^. Moreover, WES1 (GH3.5) was induced by various stress conditions such as cold, drought and heat treatment in *Arabidopsis thaliana*, and the overexpression of the *GH3.8* gene promoted pathogen resistance in rice^16,44,45^. Recent research indicated that auxin-glucose conjugation protects rice seedlings from hydroxyurea-induced DNA damage^46^.

According to these previous studies above, the broad substrate specificity of the enzymes related to auxin conjugation promotes a more flexible response to auxin homeostasis^16,34^. Therefore, it follows that TGW6 also has a broad substrate preference for auxin homeostasis to handle developmental and/or post-responses to rapid reactions toward stress. Whereas significant knowledge about the mechanism for auxin conjugation has been gained based on biochemical^34,35,39,40^ and structural biology with docking simulation^17,47^, the mechanism for auxin-conjugate hydrolase, especially auxin glycoconjugates, has remained largely unknown. Our study is the first report to explore a variety of substrates and to reveal the importance of IAA substructure for TGW6 from biochemical and structural biological viewpoints. This work will promote an understanding of the role of IAA-Glc hydrolysis for auxin homeostasis. Further, we need to confirm the interaction against TGW6 using a series of auxin conjugates such as IAA-Ala, IAA-*myo*-inositol, IBA-Glc and possible isomers of IAA-Glc etc. to reveal the comprehensive substrate specificity for auxin conjugates.

In conclusion, our study identified the structure and the substrate preference for indole fragments during substrate recognition in TGW6 based on several biochemical and computational experiments. These findings provide essential information for understanding the enzyme activity mechanism of TGW6. Revealing the details of TGW6 function will contribute to elucidating the function or role of auxin glycoconjugate hydrolysis for auxin homeostasis. In addition, future plant science investigations and biochemical and/or structural analyses will be required to accelerate the application of TGW6 for agriculturally important objectives. Designing a drug for TGW6 based on the analyzed protein structure and identifying important ligands interacting with TGW6 from the drug discovery approach will facilitate chemical control of auxin homeostasis as a novel strategy to increase crop yields.

## Methods

### Heterologous protein expression and purification

Recombinant TGW6 was expressed and purified as described previously^13^. Recombinant TGW6 with a thioredoxin and hexahistidine (Trx-(His)_6_) tag at the N-terminus was overexpressed in *Escherichia coli* Rosetta-gami 2(DE3) cells. The cells were collected and sonicated on ice in a sonication buffer (20 mM Tris–HCl, 500 mM NaCl, 5 mM imidazole, 2 mM 2-mercaptoethanol and pH 8.0). The cell lysate was centrifuged at 56,000 × *g* for 15 min at 4 °C. Recombinant TGW6 was purified by four consecutive chromatography steps using an ÄKTA Explorer system (Cytiva, Marlborough, MA). The crude protein extract was first loaded onto a HisTrap HP 5 mL column (Cytiva) for Ni-affinity chromatography. The proteins were eluted with an elution buffer containing 500 mM imidazole. Eluted TGW6 was loaded onto a HiLoad 26/60 Superdex 75 prep grade gel filtration column (Cytiva) that had been equilibrated with a gel filtration buffer (20 mM Tris–HCl, 500 mM NaCl, 2 mM CaCl_2_, 2 mM 2-mercaptoethanol and pH 7.5). Peak fractions were collected, and the Trx-(His)_6_ tag was then cleaved by incubation with thrombin (Cytiva). The protease-digested TGW6 was subjected to cation exchange chromatography using a 5 mL ToyoScreen GigaCap S 650-M column (Tosoh, Tokyo, Japan). Bound proteins were eluted by linearly increasing the concentration of NaCl from 50 to 500 mM over 20 column volumes (100 mL). Finally, peak fractions were collected and loaded onto a HiLoad 26/60 Superdex 75 prep grade column pre-equilibrated with a gel buffer (20 mM HEPES–NaOH, 150 mM NaCl, 2 mM CaCl_2_ and pH 7.5).

### X-ray crystallography

Recombinant TGW6 was purified as described above. The protein was concentrated to 6.0 mg/mL in 20 mM HEPES–NaOH, 150 mM NaCl, 2 mM CaCl_2_ and pH 7.5. Crystals of TGW6 were grown with a precipitant (0.8 M sodium citrate tribasic, 0.1 M CHES-NaOH, pH 9.6 and 3% (w/v) sucrose) at 283 K as described previously^13^. The protein crystals were collected using a nylon loop, cryoprotected by adding 25% (v/v) glycerol and then flash-cooled in liquid nitrogen. X-ray diffraction of the TGW6 crystals was conducted on beamline 26B1 with an Eiger X 4 M (Dectris) at SPring-8 (Hyogo, Japan). The wavelength was 0.999994Å, and the camera distance was 180 mm. X-ray (50 μm pinhole) exposure was for 0.3 s with a 0.1° oscillation per image^13^. The diffraction data sets were processed using XDS^48^. The phases of TGW6 were determined by molecular replacement^49^ based on the structure of strictosidine synthase 1 (PDB: 2FPB)^18^ using MOLREP^49^ in the Collaborative Computation Project Number 4 (CCP4) Program Suite v7.1.018^50^. The model structure of TGW6 was automatically built by BUCCANEER^51^ and refined using REFMAC5^52^. Finally, the structure was manually built with COOT^53^, and the crystal structure was validated using Molprobity^54^. Proteins with structural similarity to TGW6 were identified using the Protein Data Bank (PDB) with the DALI server and chain A of TGW6 as the template structure^21^. Protein graphics were created using PyMOL (version 2.5.2, Schrödinger, http://pymol.org/pymol).

For single-wavelength anomalous diffraction analysis, the protein was concentrated to 6.0 mg/mL in 20 mM HEPES–NaOH, 150 mM NaCl, 2 mM CaCl_2_ and pH 7.0. Crystals of TGW6 were grown in the presence of a precipitant (1 M sodium citrate tribasic, 0.1 M CHES-NaOH, pH 9.5, 3% (w/v) sucrose, 10 mM IAA and 2 mM CaCl_2_) at 283 K as described previously. The protein crystals were collected using a nylon loop, cryoprotected by adding 25% (v/v) glycerol containing 2 mM CaCl_2_ and then flash-cooled in liquid nitrogen. X-ray diffraction of TGW6 crystals was conducted on a beamline BL1A with an Eiger X 4 M (Dectris) at the Photon Factory (Ibaraki, Japan). The wavelength was 3.068010Å, and the camera distance was 60 mm. X-ray (50 μm pinhole) exposure was for 0.25 s with a 0.25° oscillation per image. The diffraction data were processed as described above. The phases of TGW6 were determined by molecular replacement based on the TGW6 structure that was determined as described above. The model structure of TGW6 was built as described above.

### Thermal shift assay

Thermal shift assays were performed using UNCLE (Unchained Labs, Pleasanton, CA). Recombinant TGW6 was concentrated to 27.6 μM in a buffer (20 mM HEPES–NaOH, 150 mM NaCl, 2 mM CaCl_2_ and pH 7.0). Chemical compounds were prepared as 50 mM stock solutions in dimethyl sulfoxide (DMSO). The stock solutions were diluted to 2.5 mM in DMSO. The diluted reagents were added to the protein solutions at final concentrations of 55.0 μM. The same volume of DMSO was added to the control protein solutions. Samples were incubated for 60 s every 1 °C from 15 °C to 75 °C. The emitted fluorescence from tryptophan (Trp) was monitored from 250 to 720 nm. A redshift or blueshift was detected when Trp moved to hydrophilic conditions. The fluorescence was displayed as a barycentric mean (BCM) value. Measurements were made three times to confirm reproducibility. The protein’s melting temperature (T_m_) was calculated based on Boltzmann non-linear fitting and defined as the temperature at the middle of BCM curves. When the T_m_ point increased by more than 3 °C by adding the ligand, we defined it as the T_m_ value shifted by increasing the thermostability of TGW6 in reference to the reported protocol^55^.

### ^19^F NMR fragment screening

The ^19^F NMR experiment was performed on a Bruker AV500 for fragment screening and a Bruker AV400 for the competition assay, both equipped with a 5 mm broadband fluorine observation probe. All ^19^F NMR measurements were recorded at 298 K, and the instruments were operated at a ^19^F Larmor frequency of 470 or 376 MHz.

Initially developed by Kishida Chemical (Osaka, Japan), the fluorinated building block consists of 1,505 fragment compounds. The concept for selecting compounds was reported previously^56^. Stock solutions of each fragment were prepared at a concentration of 50 mM in DMSO and stored at 4 °C. The solutions were diluted in H_2_O containing 10% (v/v) D_2_O for the lock signal (the final concentrations of the CF-, CF_2_- and CF_3_-containing molecules were 0.5, 0.25 and 0.17 mM, respectively), and chemical shifts of each compound were determined. Based on the preparation, 1,134 compounds with no impurities and high signal intensity were selected and used in subsequent experiments. Solutions of the chosen compounds were mixed using compounds whose signals did not overlap (stock mixture solution). The stock solutions of the mixtures were prepared at 2.5 mM CF-, 1.25 mM CF_2_- and 0.8 mM CF_3_-containing fragments in DMSO. These mixture solutions contained 15–18 fragments.

The final concentrations of the stock mixture solutions, CF-, CF_2_- and CF_3_-containing molecules, were diluted to 50, 25 and 17.5 μM, respectively, with and without 10 μM TGW6. Fragments of the 1,134 remaining compounds were tested in the ^19^F *R*_2_ filter experiment using the Carr–Purcell–Meibom–Gill (CPMG) scheme in the absence and presence of 10 μM TGW6. For ^19^F NMR-based fragment screening, the *R*_2_ filter experiments were recorded with the CPMG scheme26 with a time interval of 40 ms between the 180° pulses and with different total lengths. The spectra were acquired with proton decoupling using the WALTZ-65 composite pulse sequence with a 90° pulse of 80 μs during the acquisition time. Data were collected with a spectral width of 120 ppm. The acquisition and repetition times were 1.16 and 5.0 s, respectively. Samples were recorded as an average of 600 scans for each spectrum. The free induction decays were multiplied with an exponential multiplication window function with a line width of 1 Hz before the Fourier transformation. Chemical shifts were referenced to the CFCl_3_ signal in water. The decreased ratio was defined as the ratio of the signal intensity when TGW6 was added to the compound mixture compared to the signal intensity obtained without TGW6. In this study, we established the threshold for signal decay at 45%. Because this parameter is influenced by background noise the threshold should be more than twice the value of the noise signal intensity. For the competition assay, fragments in the chemical mixtures containing PK0-38511 and CK0-00681 were further tested by measurements with or without 10 μM TGW6 and 10 μM TGW6 + 100 μM IAA-Glc in the same conditions as described above.

### Computational analysis

The Pipeline Pilot (v2018) (developed by Dassault, Veĺizy-Villacoublay, France) was used to process chemical data of the fragment library^57^. Fragments were characterized by calculation using a fingerprint: Pharmacophore Fingerprint, PHFP. The R program (v3.4.4) was used for clustering analysis using the single-linkage clustering method (hclust function)^58^ and the distance based on the Tanimoto coefficient. Docking simulation was performed using Flare (v6.1.0.) (Cresset, Cambridgeshire, U.K.)^59^ based on the Lead Finder, which is equipped with a dedicated algorithm and scoring function for virtual screening. A detailed description of the Lead Finder scoring function was reported previously^60^. The crystal structure of chain A of TGW6 was used as the structure of the target protein. The chemical structure of each ligand was obtained from PubChem (https://pubchem.ncbi.nlm.nih.gov). The docking grid definition was determined around the pocket detected by Flare, and the pocket with the highest druggability score, which is the benchmark score of the interaction with drug-like small molecules, was selected. The calculation mode of docking in Flare was chosen as “very accurate but slow,” and the maximum number of poses was set to 10. A docking pose was chosen for each ligand compound based on the LF Rank score, a value demonstrated by the benchmark to be suitable for comparing different docking poses of the same ligand.

The MD simulation was carried out using Flare, which is based on the OpenMM package with Amber GAFF as the force fields and AM1-BCC as the charging method. The calculation method was selected by OpenFF and Explicit Water. The solvent system was constructed by the Explicit TIP3P solvent–water model by keeping the orthorhombic shape of the box within the dimensions of 10 × 10 × 10 Å. The protein–ligand complex was later minimized by setting the minimum energy tolerance at 0.25 kcal/mol and equilibrated for 200 ps before initiating the MD run. The simulation was performed with a time step of 4.00 fs, and the simulation length was set at 10 ns.

### Supplementary Information

Supplementary Information.

## Data Availability

The accession number for the coordinates and structure factors reported in this study is PDB 8KG3 (10.2210/pdb8KG3/pdb).
