# Supplementary material for: THOUSAND-GRAIN WEIGHT 6, which is an IAA-glucose hydrolase, preferentially recognizes the structure of the indole ring
Source: Sci Rep. 2024 Mar 21;14:6778. doi: 10.1038/s41598-024-57506-z (PMC10958001; doi:10.1038/s41598-024-57506-z)
Supplement: Supplementary file 1 — Supplementary Information. [file 41598_2024_57506_MOESM1_ESM.pdf]

THOUSAND-GRAIN WEIGHT 6, which is an IAA-glucose Hydrolase, Preferentially Recognizes the Structure of the Indole Ring

Tatsuki Akabane[1], Nobuhiro Suzuki[2], Kazuyoshi Ikeda[3,4], Tomoki Yonezawa[4], Satoru Nagatoishi[5], Hiroyoshi Matsumura[6], Takuya Yoshizawa[6], Wataru Tsuchiya[2], Satoshi Kamino[7], Kouhei Tsumoto[5], Ken Ishimaru[8], Etsuko Katoh[9,*] and Naoki Hirotsu[1*]

1. Graduate School of Life Sciences, Toyo University, 1-1-1 Izumino, Itakura, Oura, Gunma 374-0193, Japan.
2. Research Center for Advanced Analysis, National Agriculture and Food Research Organization, 2-1-2 Kannondai, Tsukuba, Ibaraki 305-8518, Japan.
3. Medicinal Chemistry Data Intelligence Unit, Drug Development Data Intelligence Platform Group, Medical Sciences Innovation Hub Program (MIH), RIKEN, 1-7-22 Suehiro-cho, Tsurumi-ku, Yokohama City, Kanagawa, 230-0045, Japan.
4. Division of Physics for Life Functions, Keio University Faculty of Pharmacy, 1-5-30 Shibakoen Minato-ku, Tokyo 105-8512, Japan.
5. School of Engineering, The University of Tokyo, 7-3-1 Hongo, Bunkyo-ku, Tokyo 113-8656, Japan
6. Department of Biotechnology, College of Life Sciences, Ritsumeikan University, 1-1-1 Noji-Higashi, Kusatsu, Shiga 525-8577, Japan.
7. CRYO SHIP Incorporated, 1-266-3, Sakuragi-cho, Omiya-ku, Saitama, Saitama, 330-0854, Japan
8. Institute of Crop Science, National Agriculture and Food Research Organization, 2-1-2 Kannondai, Tsukuba, Ibaraki 305-8518, Japan.
9. Department of Food and Nutritional Sciences, Toyo University, 1-1-1 Izumino, Itakura, Oura, Gunma, 374-0193, Japan.

Table S1. Proteins that have structural similarity with TGW6 based on the DALI server

Name	PDB ID	Function	Z score	RMSD (Å)	Length of alignment (residue)	No. of residues	Sequence identity (%)
STR1	2FP8	lyase	41.1	1.7	288	303	31
DFPase	1PJX	hydrolase	29.8	2.6	267	314	21
XC5397	3DR2	hydrolase	27.5	2.7	251	299	17
Drp35	2DG1	hydrolase	26.7	2.8	261	321	21
NHL domain of TRIM71	6FPT	RNA binding	26.3	2.7	253	391	15
PON1	1V04	hydrolase	26.3	3.3	267	332	14
PAL	3FVZ	lyase	23.6	3.1	257	329	14
Mala s 1	2P9W	allergen	23.3	2.9	250	333	11
apo-form of PDH	6JT5	oxidoreductase	22.8	3.0	254	409	13
LRE	5D9B	hydrolase	22.7	3.1	260	307	15

Z-score is a similarity score of the hit structure against the query structure. RMSD (Å) is Root Mean Square Deviation of the atomic positions in a three-dimensional structure. Length of alignment (residue) is the number of aligned amino acids. No. of residues is the number of residues in the protein. Sequence identity (%) is the percent of sequence identity. Strictosidine synthase (STR1) (PDB: 2FP8), diisopropylfluorophosphatase (DFPase) (PDB: 1PJX), gluconolactonase (XC5397) (PDB: 3DR2), drug resistance protein 35 (DRP35) (PDB: 2DG1), the NHL domain of E3 ubiquitin-protein ligase tripartite motif-containing protein 71 (NHL domain of TRIM71) (PDB: 6FPT), paraoxonase (PON1) (PDB: 1V04), peptidyl-alpha-hydroxyglycine alpha-amidating lyase (PAL) (PDB: 3FVZ), *Malassezia sympodialis* allergen (Mala s 1) (PDB: 2P9W), pyranose dehydrogenase (PDL) (PDB: 6JT5) and luciferin-regenerating enzyme (LRE) (PDB: 5D9B)

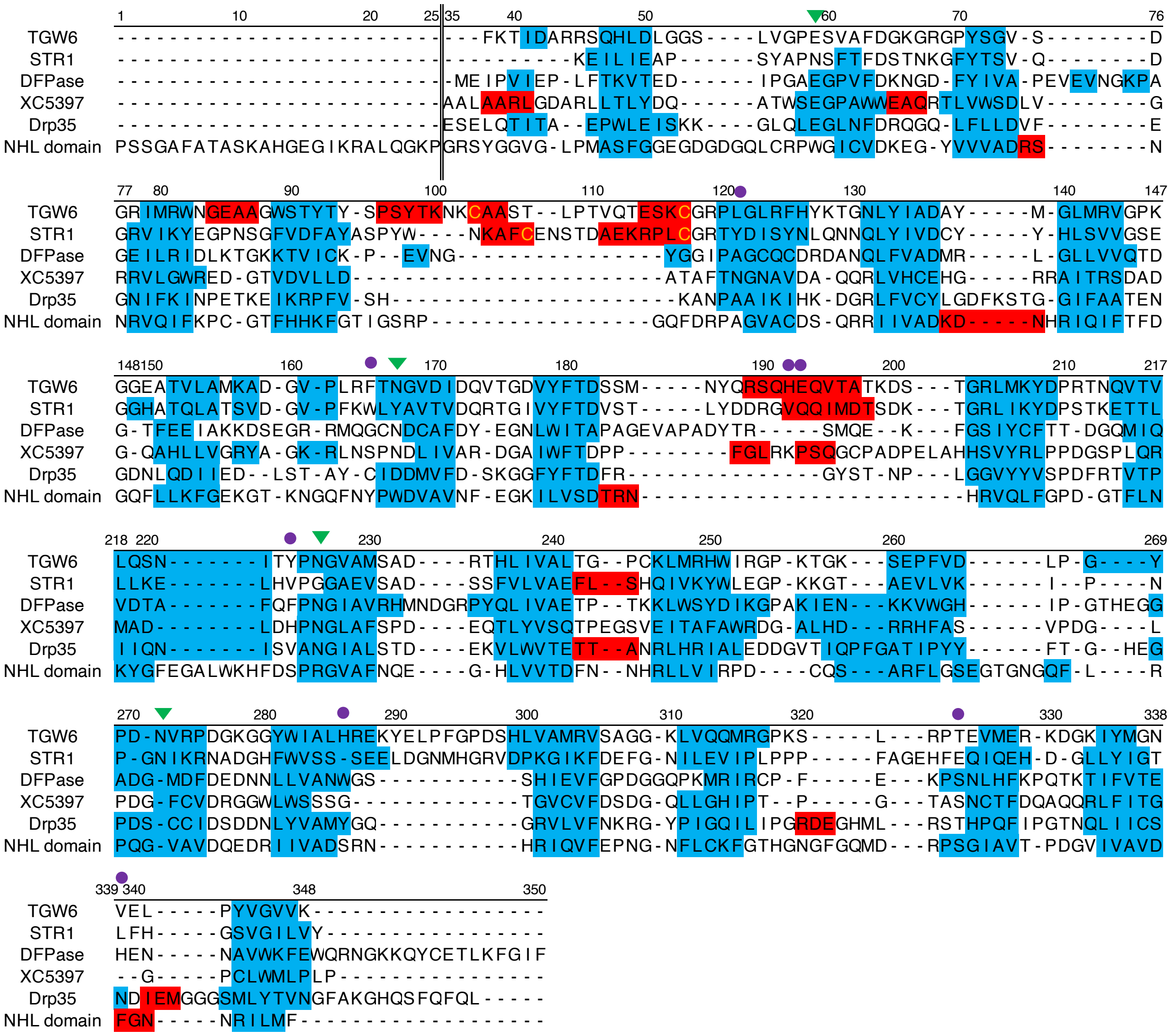

Figure S1. Amino acid sequence alignment of structurally similar proteins and TGW6

Amino acids shown in blue and red are the residues constituting the β-strand or α-helix, respectively. Cysteine residues shown in yellow create the S-S bond. The inverted green triangles and purple dots identify residues of the conserved Ca^{2+}-binding site and putative active site, respectively.

Table S2. Crystallographic data collection and refinement statistics of TGW6 in anomalous diffraction

**PF Beam line**	**BL-1A**
Diffraction Data	
Wavelength (Å)	3.06801
Temperature	100 K
Crystal-detector distance (mm)	60
Rotation range per image	0.25°
Exposure time per image (sec)	0.25
Space group	*H 3 2*
Cell parameters	a = 216.67 Å, b = 216.67 Å, c = 246.58 Å
Resolution range (Å)	49.77-3.24 (3.40-3.24)
No. of reflections	603,784 (50.152)
No. of unique reflections	35,185 (4,389)
Completeness (%)	99.3 (94.7)
R_{merge} (%)	19.8 (75.6)
Redundancy	17.2 (11.4)
I/σ (I)	14.0 (2.9)
CC (1/2)	99.4 (87.2)
Refinement and structure model	
R_{work} / R_{free} factor	0.205 / 0.233
No. of molecules	3
Average B-factor (Å^2)	70.93
RMSD	
Bond length (Å)	0.0133
Bond angles (°)	1.67
Ramachandran plot (%)	
Favored	94.28
Allowed	4.96
Outliers	0.76

Values in parentheses are for the highest-resolution shell.

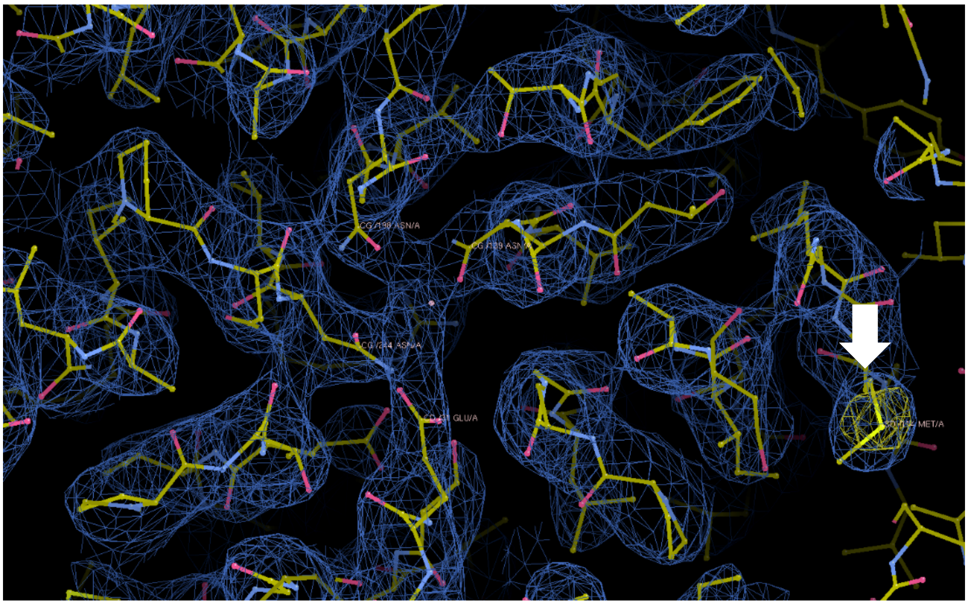

Figure S2. Electron density map of TGW6 that was obtained from the anomalous diffraction experiment

The modeled crystal structure of TGW6 is indicated as yellow sticks in the blue mesh. The yellow mesh surrounding the sulfur of the methionine residue at the right most part of the figure that is pointed by a white arrow indicates the electron density map from anomalous diffraction. The sulfur ion peak that also causes anomalous diffraction by irradiating relatively near an X-ray wavelength.

a: Control

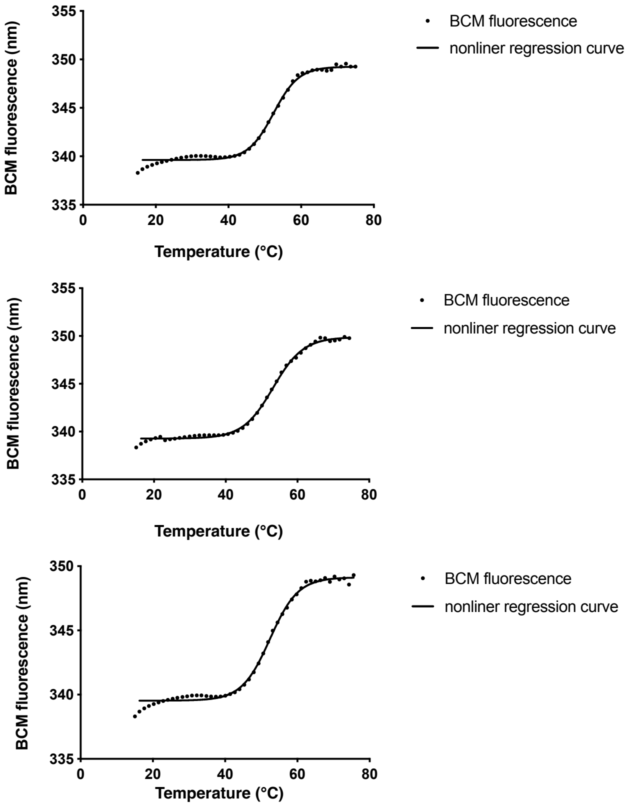

b: IAA-Glc

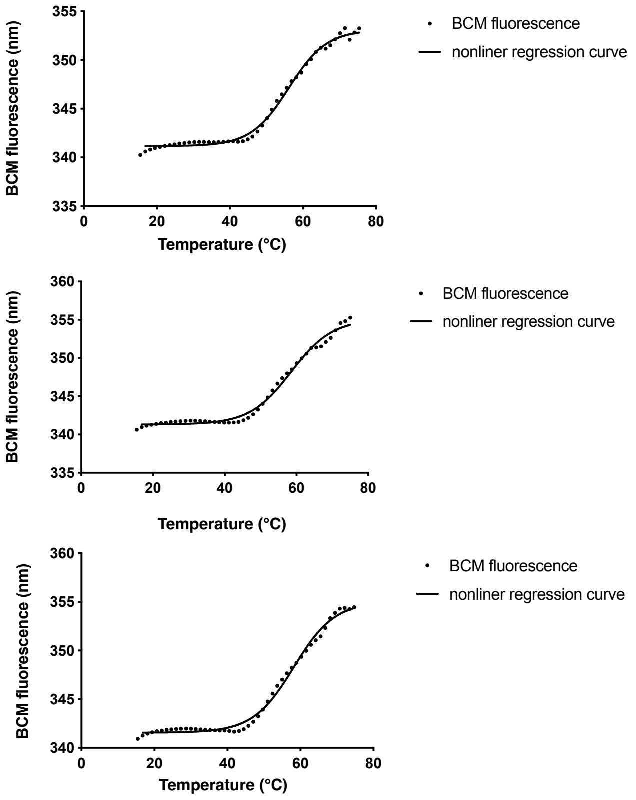

c: IAA-Ala

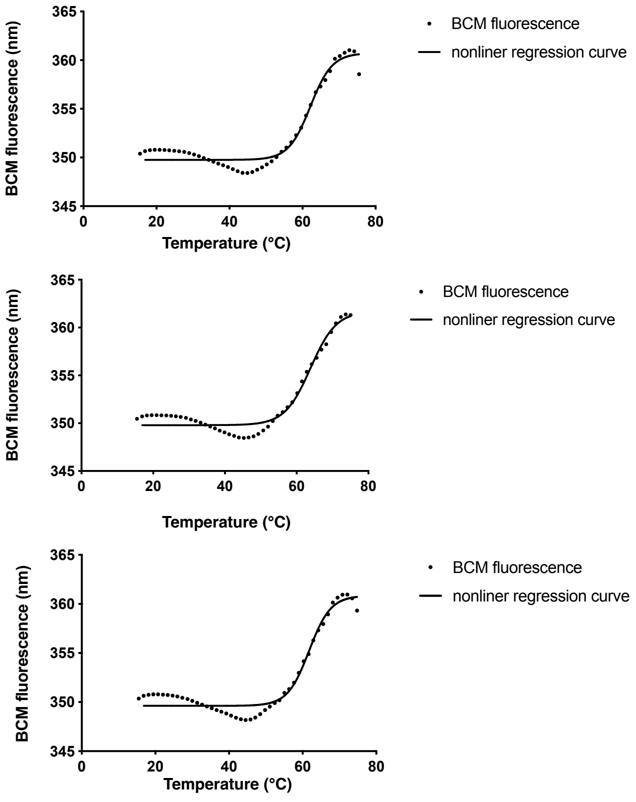

d: IAA

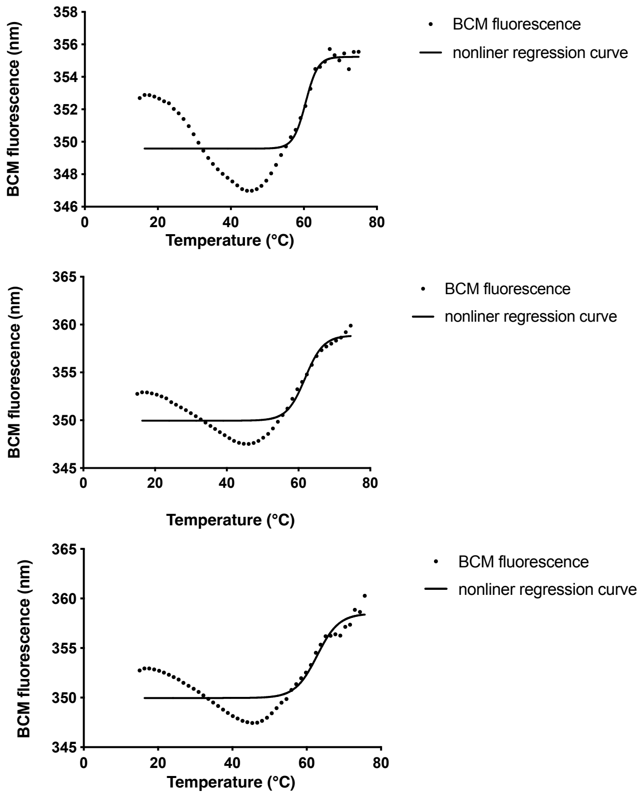

e: IBA

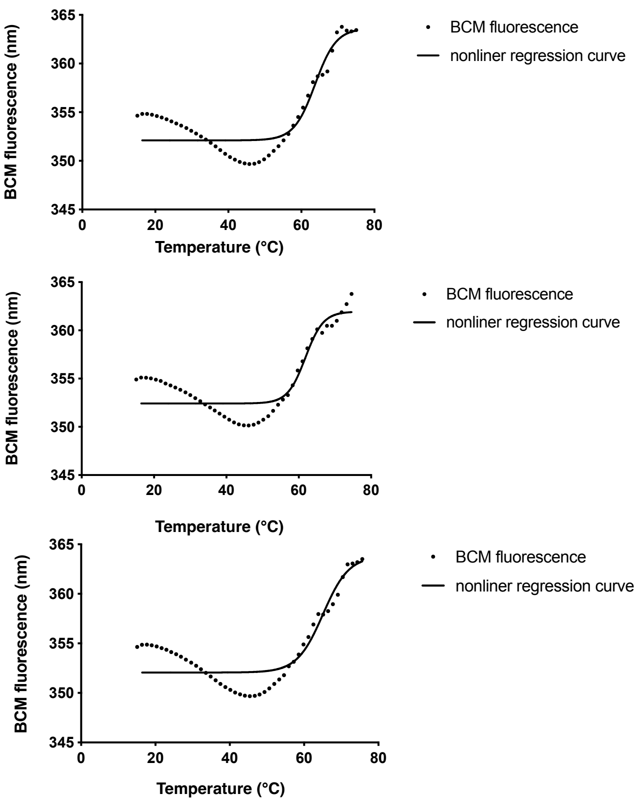

f: MeIAA

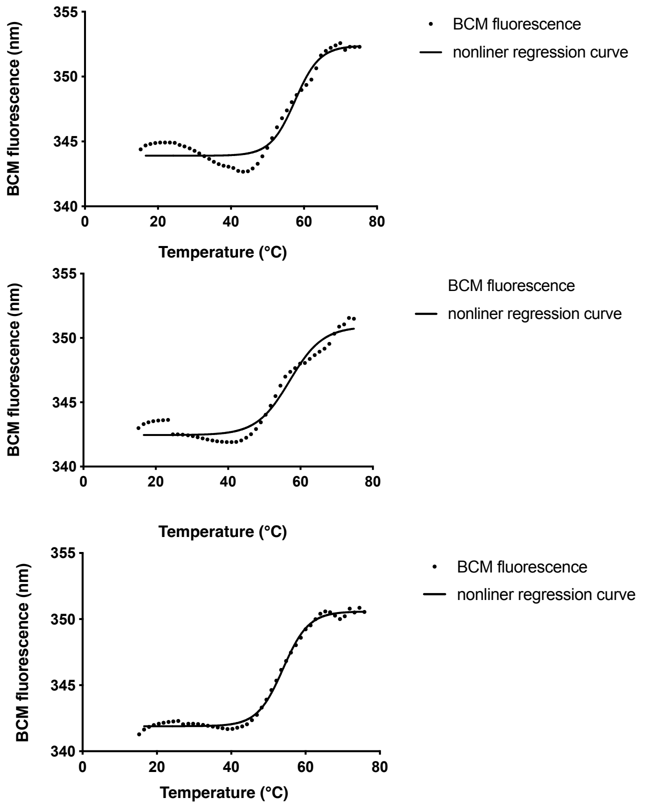

g: IPA

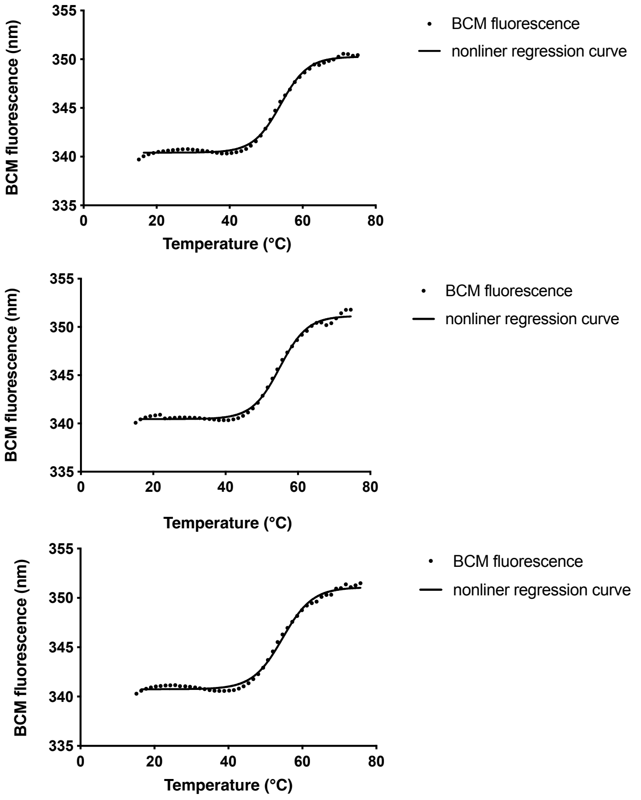

h: oxIAA

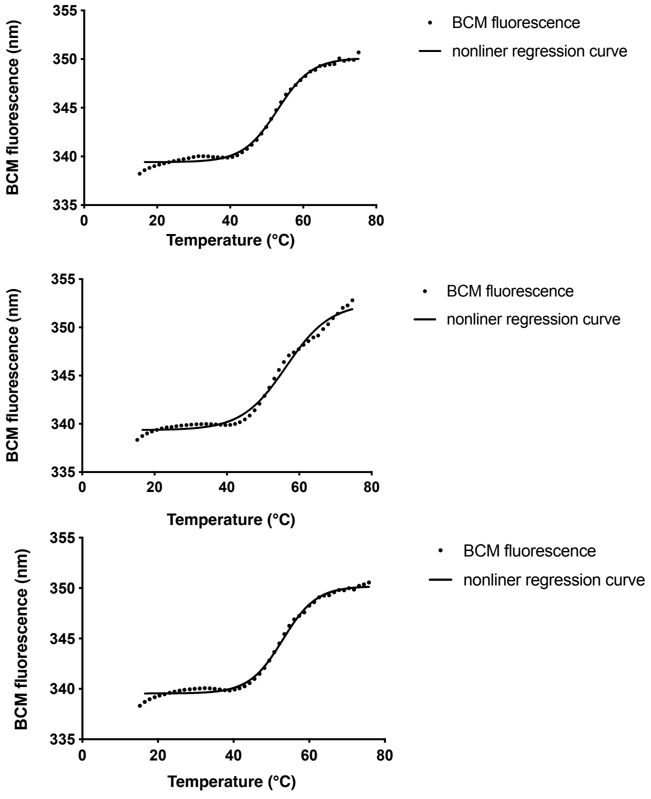

i: 4-Cl-IAA

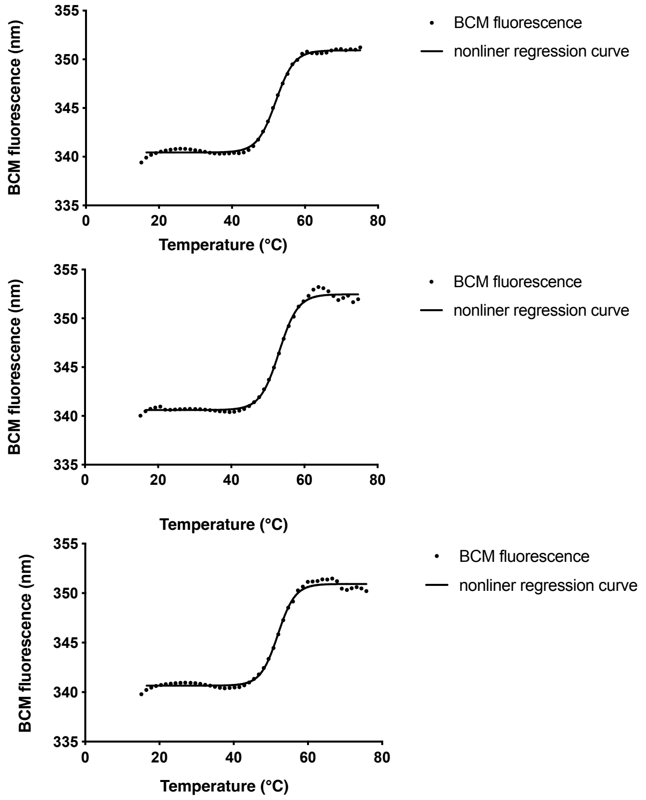

j: ICA

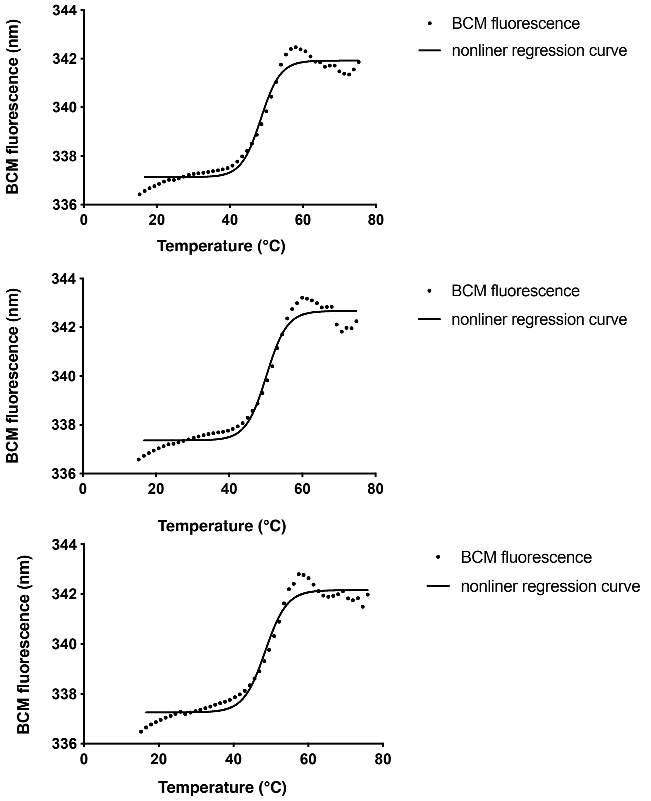

k: Glc

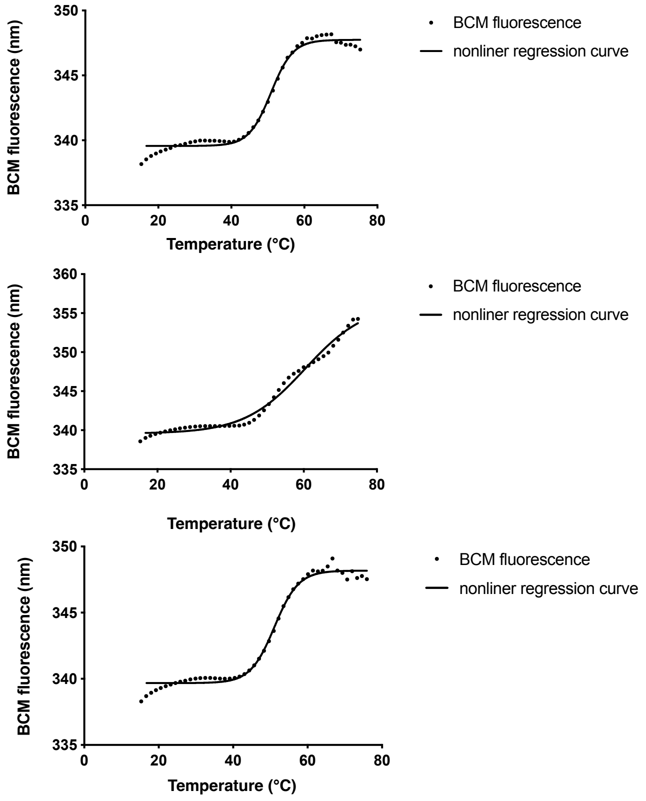

l: Inositol

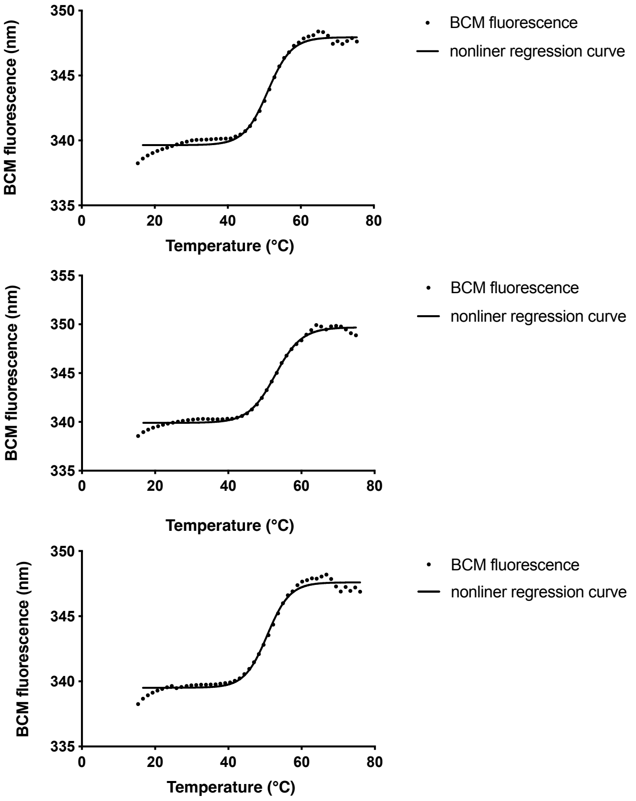

m: UDP-Glc

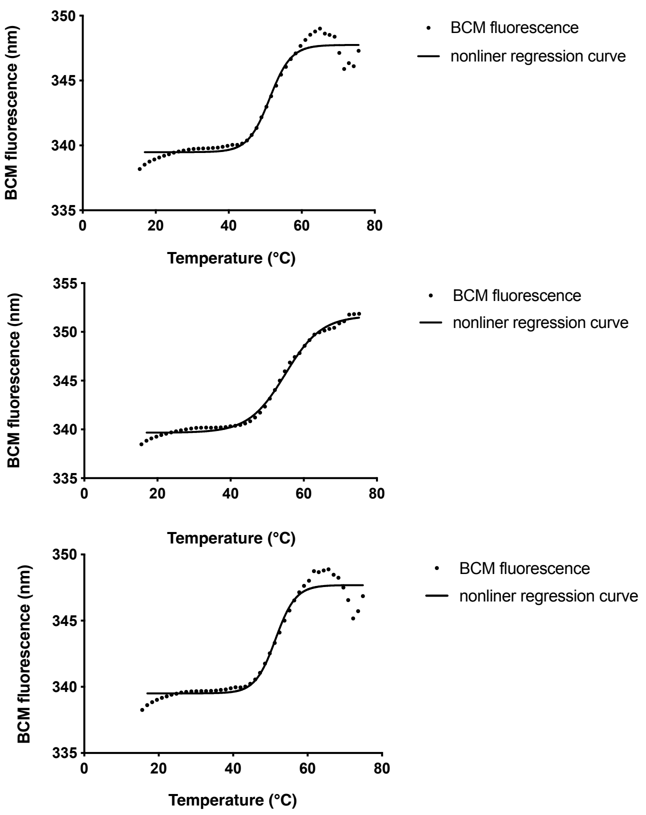

n: p-n-b-Glc

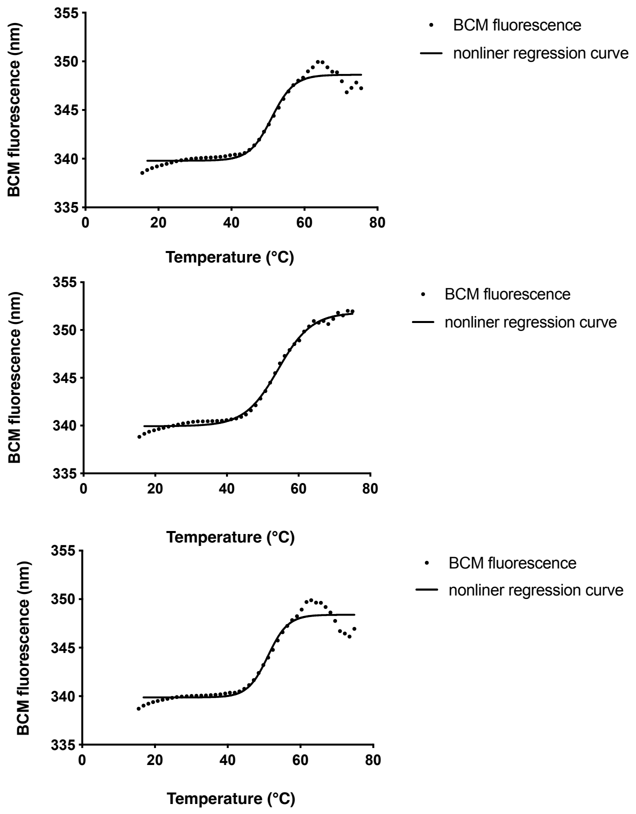

o: Galactinol

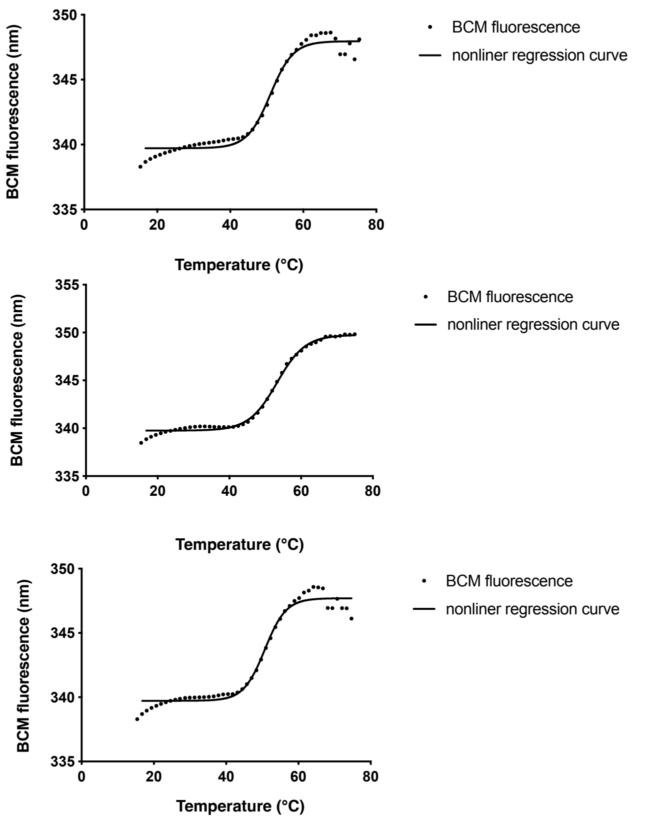

p: PK0-38511

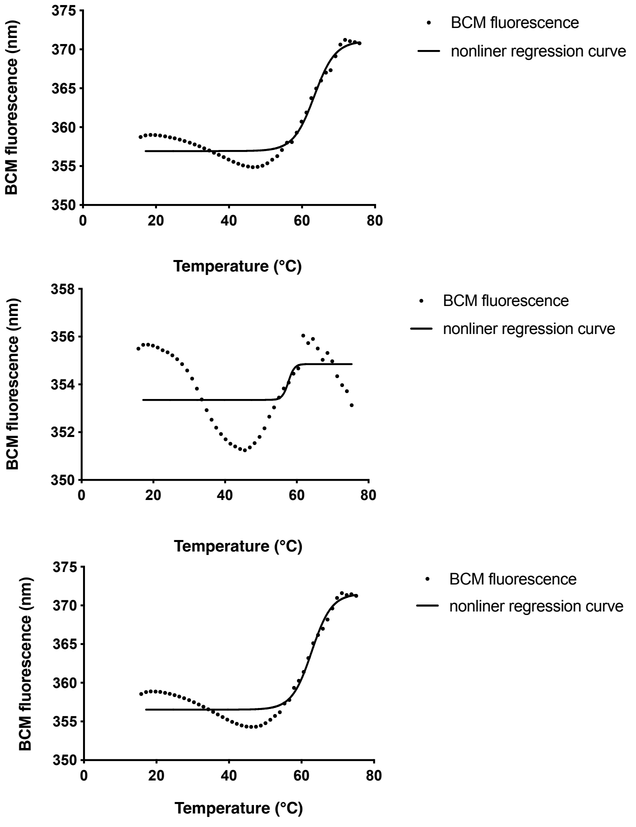

q: PK0-38511

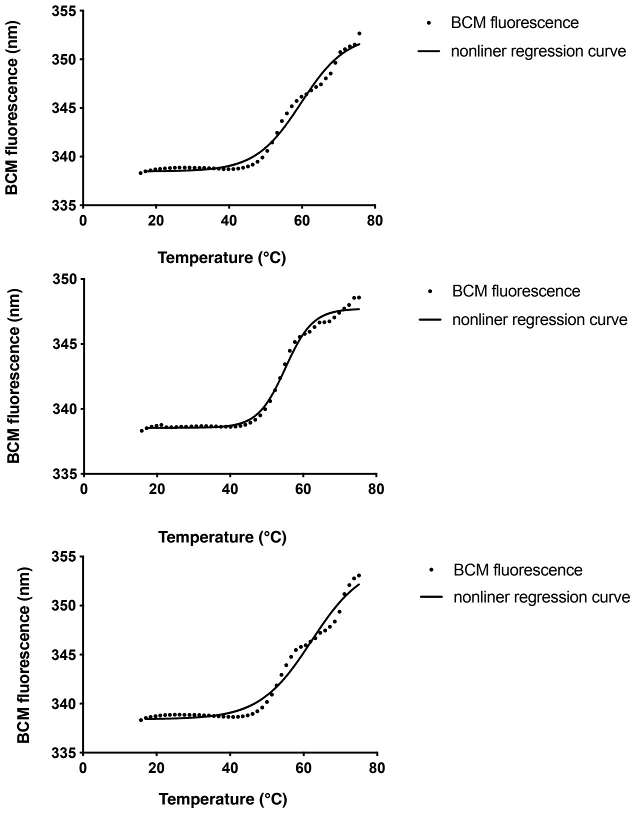

r: PK0-42071

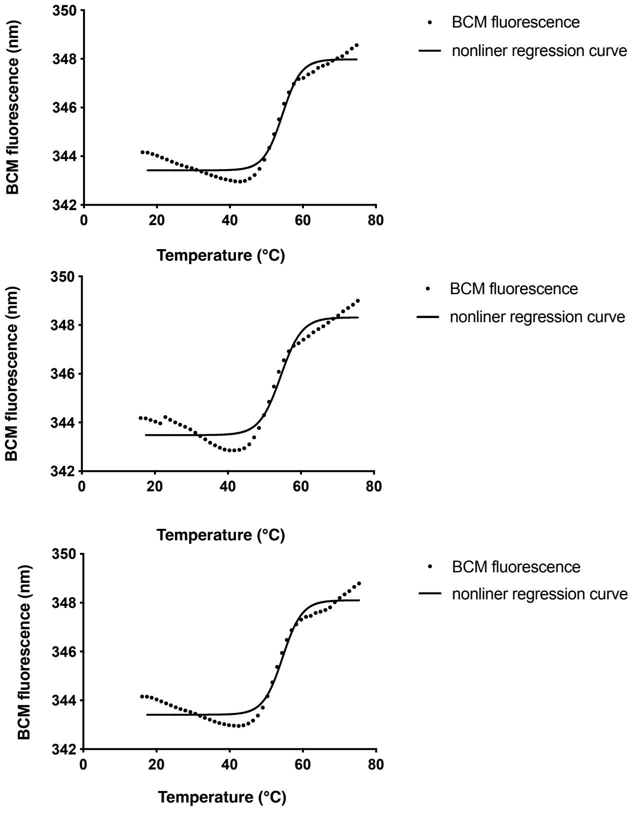

s: CK0-00681

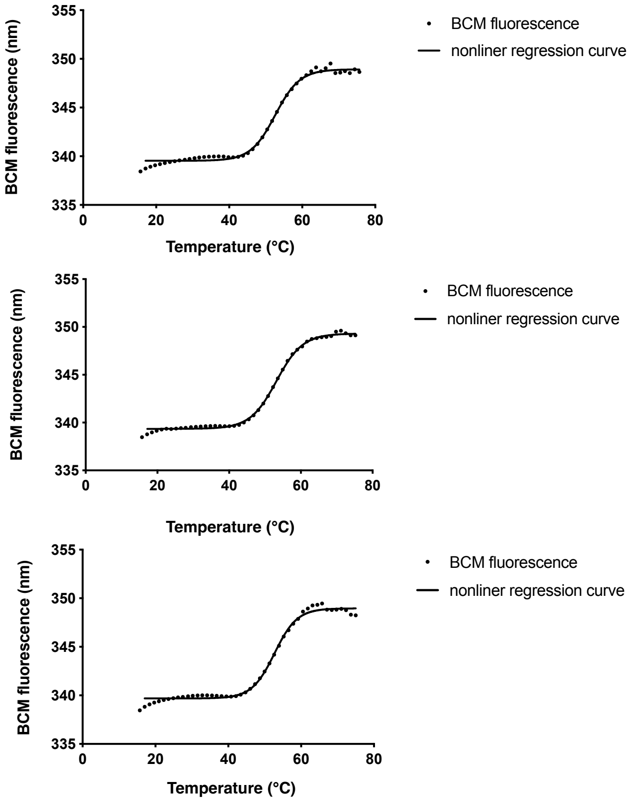

t: CK0-02991

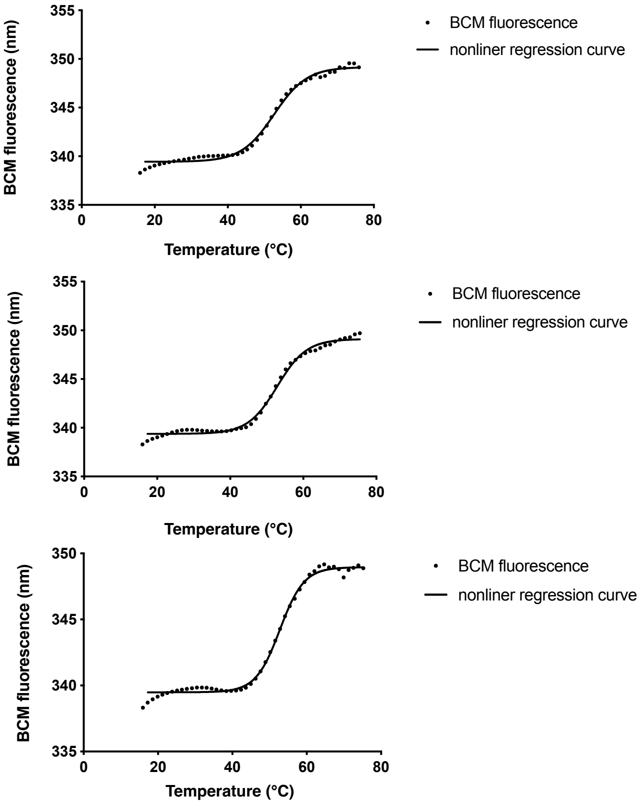

u: CK0-03011

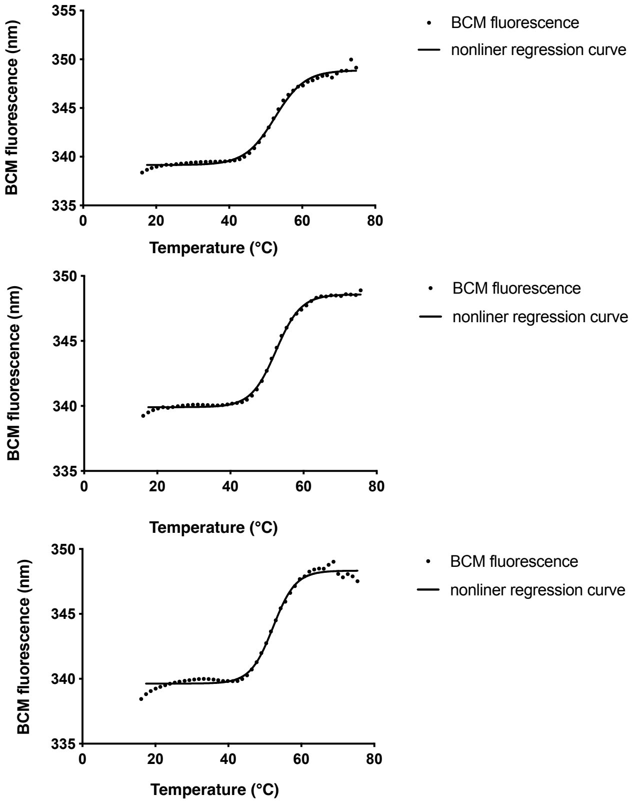

Figure S3. BCM fluorescence curves of TGW6 with or without each ligand

Plots of the wavelength of BCM fluorescence from TGW6 and the non-linear regression curve of the plot in the presence of DMSO (a), IAA-Glc (b), IAA-Ala (c), IAA (d), IBA (e), MeIAA (f), IPA (g), oxIAA (h), 4-Cl-IAA (i), ICA (j), Glc (k), Inositol (l), UDP-Glc (m), p-n-b-D-Glc (n), Galactinol (o), PK0-38501 (p), PK0-38511 (q), PK0-42071 (r), CK0-00681 (s), CK0-02991 (t) and CK0-00311 (u). Three measurements were made to confirm reproducibility, and each figure documents the obtained results, respectively. For the measurements, the final concentration of recombinant TGW6 was adjusted to 27.6 µM with a 55.0 µM ligand solution.

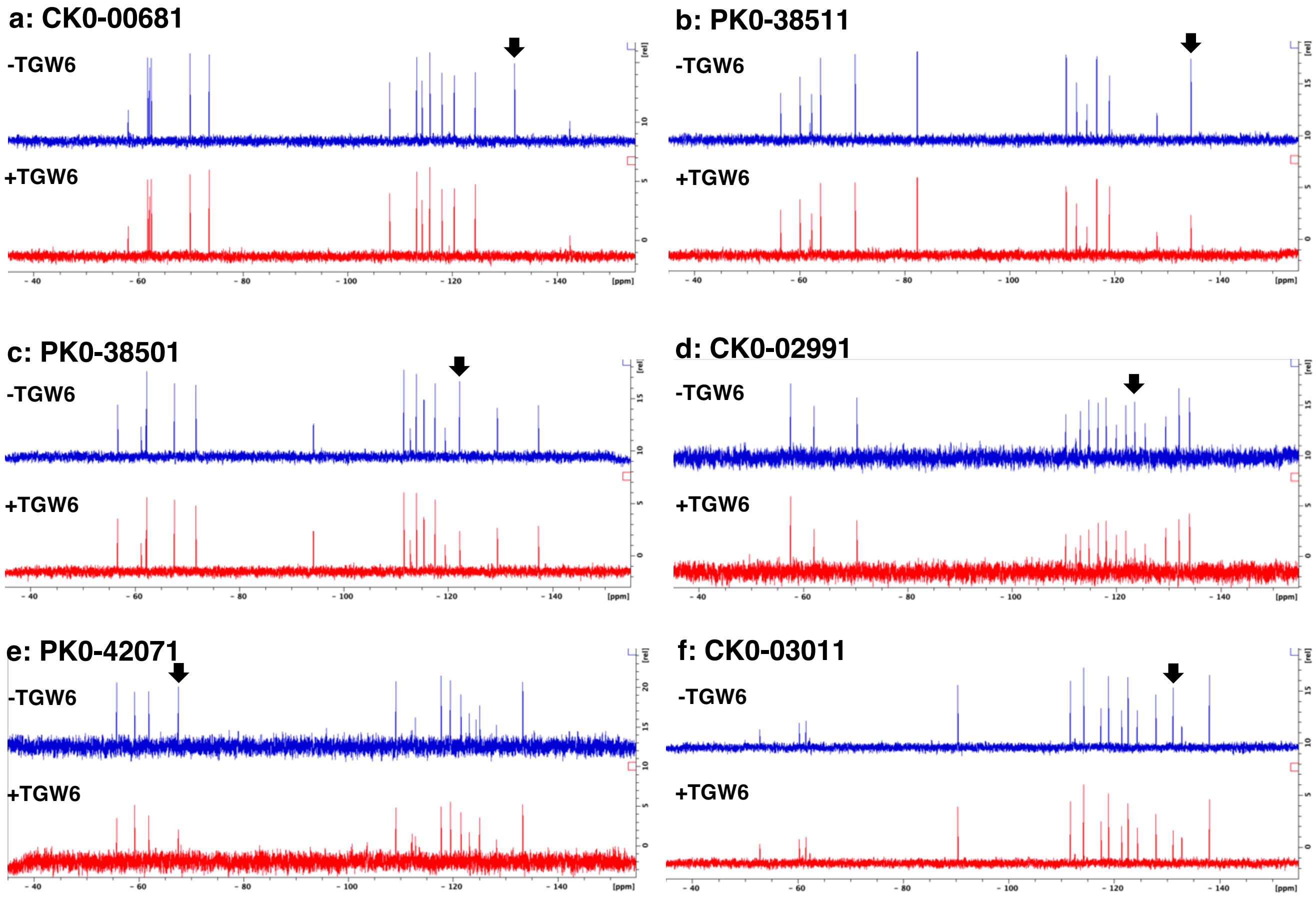

Figure S4. Representative ^{19}F NMR spectra of a mixture of fluorine-containing compounds in the absence and presence of TGW6

^{19}F NMR spectra of a chemical mixture with TGW6 (red) and without TGW6 (blue). The solution contained 16 fragments, and the black downward arrow points to the ^{19}F NMR signal of CK0-00681 at -131.95 ppm (a); The solution contained 17 fragments, and the black downward arrow points to the ^{19}F NMR signal of PK0-38511 at -134.55 ppm, (b); The solution contained 16 fragments, and the black downward arrow points to the ^{19}F NMR signal of PK0-38501 at -122.21 ppm, (c); The solution contained 17 fragments, and the black downward arrow points to the ^{19}F NMR signal of CK0-02991 at -123.54 ppm, (d); The solution contained 12 fragments, and the black downward arrow points to the ^{19}F NMR signal of PK0-42071 at -67.56 ppm, (e); The solution contained 17 fragments, and the black downward arrow points to the ^{19}F NMR signal of CK0-03011 at -131.19 ppm, (f).

Table S3. Signal decrease ratio of fragments that belong to the same pharmacophore cluster with hit fragments

ID	Chemical Name	Intensity △(%)	cluster No. PFFP
CK0-00681	2-[(2-Fluoro-4-nitrophenoxy)methyl]tetrahydrofuran	-100	47
PK0-11341	3-(2-Fluoro-4-nitrophenoxy)oxetane	-32.3	47
CK0-00761	2-Fluoro-1-isopropoxy-4-nitrobenzene	-17.4	47
CK0-02991	1-(3-Fluoro-2-nitrophenyl)piperidin-4-ol	-57.9	29
CK0-03271	4-(2-Fluoro-6-nitrophenoxy)piperidine hydrochloride	-18.1	29
CK0-02821	4-(3-Fluoro-2-nitrophenoxy)piperidine hydrochloride	-14.6	29
CK0-02111	N-(3-Fluoro-2-nitrophenyl)piperidin-4-amine hydrochloride	-9.6	29
CK0-02021	1-(3-Fluoro-2-nitrophenyl)piperidin-4-amine hydrochloride	-2.2	29
CK0-02161	N-(2-Fluoro-6-nitrophenyl)piperidin-4-amine hydrochloride	0.8	29
CK0-02031	1-(4-Fluoro-2-nitrophenyl)piperidin-4-amine hydrochloride	1.7	29
CK0-02191	N-(4-Fluoro-2-nitrophenyl)piperidin-4-amine hydrochloride	11.8	29
CK0-03371	4-(4-Fluoro-2-nitrophenoxy)piperidine hydrochloride	13.4	29
CK0-03011	4-(2-Fluoro-4-nitrophenoxy)tetrahydro-2H-pyran	-45.4	48
CK0-03591	4-(2-Fluoro-4-nitrophenoxy)piperidine hydrochloride	1.3	48
PK0-38511	2-(7-Fluoro-1H-indol-3-yl)acetic acid	-50.3	264
PK0-38501	2-(4-Fluoro-1H-indol-3-yl)acetic acid	-46.5	264
PK0-42071	3,3,3-Trifluoro-2-(5-fluoro-1H-indol-3-yl)propanoic acid	-46.0	264
PK0-41071	3,3,3-Trifluoro-2-(5-methyl-1H-indol-3-yl)propanoic acid	-33.4	264
PK0-41421	3,3,3-Trifluoro-2-(7-fluoro-1H-indol-3-yl)propanoic acid	-16.9	264
PK0-50391	2-[5-(Trifluoromethyl)-1H-indol-3-yl]acetic acid	-14.7	264
PK0-49291	2-[6-(Trifluoromethyl)-1H-indol-3-yl]acetic acid	-11.6	264
PK0-39401	3,3,3-Trifluoro-2-(7-methyl-1H-indol-3-yl)propanoic acid	-10.4	264
PK0-39391	3,3,3-Trifluoro-2-(6-methyl-1H-indol-3-yl)propanoic acid	-9.3	264
PK0-39381	3,3,3-Trifluoro-2-(1H-indol-3-yl)propanoic acid	-8.8	264
PK0-41411	3,3,3-Trifluoro-2-(6-fluoro-1H-indol-3-yl)propanoic acid	-6.4	264
PK0-49411	2-[4-(Trifluoromethyl)-1H-indol-3-yl]acetic acid	-0.1	264

ID indicates the ID number of the fluorine fragment in the chemical library (Kishida Chemical). ΔIntensity is the reduction percentage of the NMR signal intensity in the ^{19}F NMR fragment screening. Cluster No. PHFP is the cluster number based on the pharmacophore fingerprint.

Table S4. Competition assay of hit fragments containing an indole ring or another functional group in the presence of IAA-Glc

ID	Δ Intensity (%)	Recovery ratio with IAA-Glc (%)
CK0-00681	-100	0
PK0-38511	-43.5	28.5

ID indicates the ID number of the fluorine fragment in the chemical library (Kishida Chemical).

Δ Intensity is the reduced percentage of the NMR signal intensity in the ^{19}F NMR fragment screening.

The recovery ratio with IAA-Glc (%) is the recovery of the NMR signal intensity after adding IAA-Glc. This value was calculated from the reduced signal intensity in the presence of TGW6 as the basement, and the intensity of the control condition as the maximum.

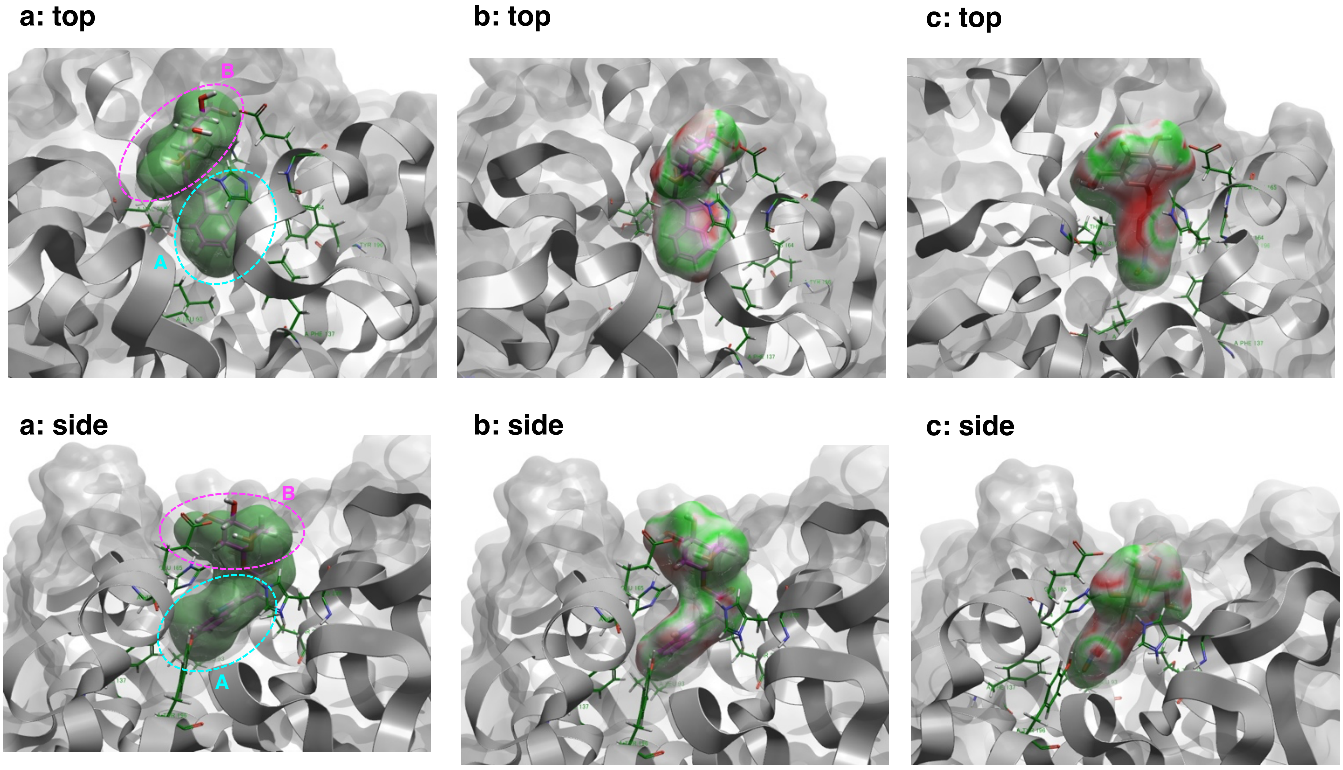

Figure S5. Docking pose of IAA-Glc against TGW6 with the detected pocket (a), the electrostatic complementarity (b) and the electrostatic complementarity of p-n-b-D-Glc (c)

(a) A portion of TGW6 is represented as a drawing and surface model shown in gray. IAA-Glc is shown as purple sticks. The green object is the detected pocket of TGW6. The interacting residues are represented as green sticks. The dashed circle in cyan is subpocket A and the magenta dashed circle is subpocket B. (b) A portion of TGW6 and IAA-Glc are represented in the same manner in (a). The interacting residues are represented as green sticks. The object that is colored in green and red indicates electrostatic complementarity. The green colored region shows that the electrostatic surface is a relative match. The red colored region indicates that the electrostatic surface is relatively repelled. (c) A portion of TGW6 is represented in the same manner as in (a). p-n-b-D-Glc is indicated as brown sticks. The interacting residues for p-n-b-D-Glc are represented as green sticks. The object that is colored in green and red indicates electrostatic complementarity. The green colored region shows that the electrostatic surface is a relative match. The red colored region indicates that the electrostatic surface is relatively repelled.

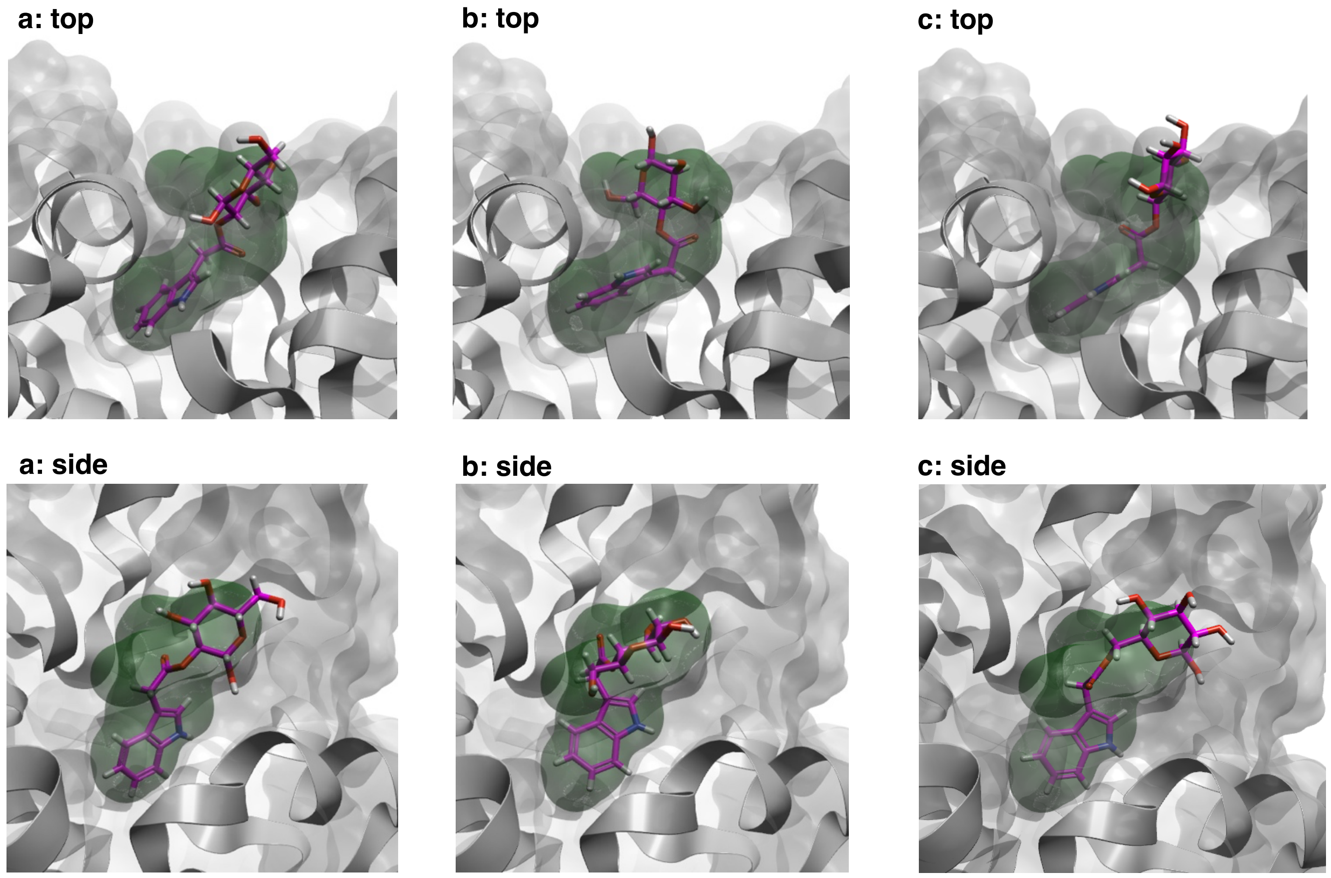

Figure S6. Docking pose of IAA-Glc structural isomers in the presence of TGW6 with the detected pocket

A portion of TGW6 is schematically represented as a gray surface model. IAA-Glc is shown as a purple stick model. The green object is the detected pocket of TGW6. 2-*O*-IAA-Glc, (a); 4-*O*-IAA-Glc, (b); 6-*O*-IAA-Glc, (c).

Table S5. Residues in contact with the docked IAA-Glc in the presence of TGW6

Bond Type	Ligand Atom	Protein Atom	Frames Present
Hydrogen bond	H5	Glu-340 OE1	66.00%
Hydrogen bond	H7	Glu-340 OE1	59.60%
Hydrophobic	C13	Arg-189 CG	53.00%
Hydrophobic	C15	Phe-165 CE1	44.50%
Hydrophobic	C13	Phe-165 CE1	43.10%
Hydrogen bond	H9	Arg-189 O	41.50%
Hydrophobic	C12	Thr-324 CG2	41.00%
Hydrogen bond	H5	Glu-340 OE2	38.10%
Hydrogen bond	H7	Glu-340 OE2	33.80%
Aromatic-Aromatic	H15	His-286 CD2	31.90%
Hydrophobic	C13	Phe-165 CZ	28.60%
Hydrogen bond	H8	Glu-193 OE1	26.70%
Hydrophobic	C7	Val-339 CG2	25.90%
Cation-pi	C11	Arg-189 NH1	20.50%
Aromatic-Aromatic	H14	His-192 CD2	19.70%
Hydrophobic	C8	Val-339 CG2	18.70%
Aromatic-Aromatic	C11	His-192 CD2	17.60%
Hydrophobic	C15	Phe-165 CD1	17.40%
Hydrophobic	C7	Thr-324 CG2	16.60%
Hydrogen bond	O5	Arg-287 HH21	14.70%
Hydrophobic	C14	Asn-167 CB	13.40%
Hydrophobic	C13	Phe-165 CD1	12.20%
Hydrophobic	C14	Thr-324 CG2	8.60%
Hydrogen bond	H8	Glu-193 OE2	8.10%
Hydrogen bond	O6	Arg-322 HH21	8.10%
Hydrogen bond	O	His-192 HE2	8.00%
Hydrophobic	C9	Val-339 CG1	7.90%
Hydrophobic	C7	His-286 CB	7.90%
Hydrogen bond	O4	Arg-322 HH22	7.80%
Hydrophobic	C8	Val-339 CG1	7.30%
Hydrogen bond	O6	Asn-338 HD22	6.60%
Hydrophobic	C15	Phe-165 CZ	6.40%
Hydrophobic	C15	Asn-167 CB	6.10%

~continuation of Table S5~

Bond Type	Ligand Atom	Protein Atom	Frames Present
Hydrophobic	C7	Val-339 CG1	6.10%
Hydrogen bond	H9	His-192 ND1	5.00%
Hydrophobic	C15	Arg-189 CG	4.30%
Hydrogen bond	O5	His-192 HE2	3.80%
Hydrogen bond	O3	Arg-322 HH11	3.50%
Hydrophobic	C13	His-192 CB	3.10%
Hydrogen bond	O3	Arg-322 HE	2.70%
Hydrogen bond	H6	Glu-340 OE1	2.50%
Hydrophobic	C9	Thr-324 CG2	2.50%
Cation-pi	C15	Arg-189 NH1	2.30%
Hydrogen bond	O3	Arg-322 HH21	2.20%
Hydrogen bond	O5	Arg-287 HH22	1.80%
Hydrogen bond	O3	Arg-322 HH22	1.70%
Hydrophobic	C12	Val-339 CG1	1.60%
Hydrophobic	C13	Val-339 CG1	1.50%
Hydrogen bond	O5	His-286 HE2	1.50%
Hydrophobic	C14	Phe-165 CE1	1.30%
Aromatic-Aromatic	C15	Phe-165 HE1	1.30%
Hydrogen bond	O1	His-192 HE2	1.10%
Hydrogen bond	O	His-286 HE2	1.10%
Hydrogen bond	H8	Arg-287 NE	1.10%

Bond type, the ligand-protein interaction of IAA-Glc and TGW6; Ligand atom, the position of the atom in IAA-Glc contacting TGW6; Protein atom, the position of the atom in TGW6 interacting with IAA-Glc; Frames present, the percentage of time during which interaction existed throughout a simulation period of 10 ns.

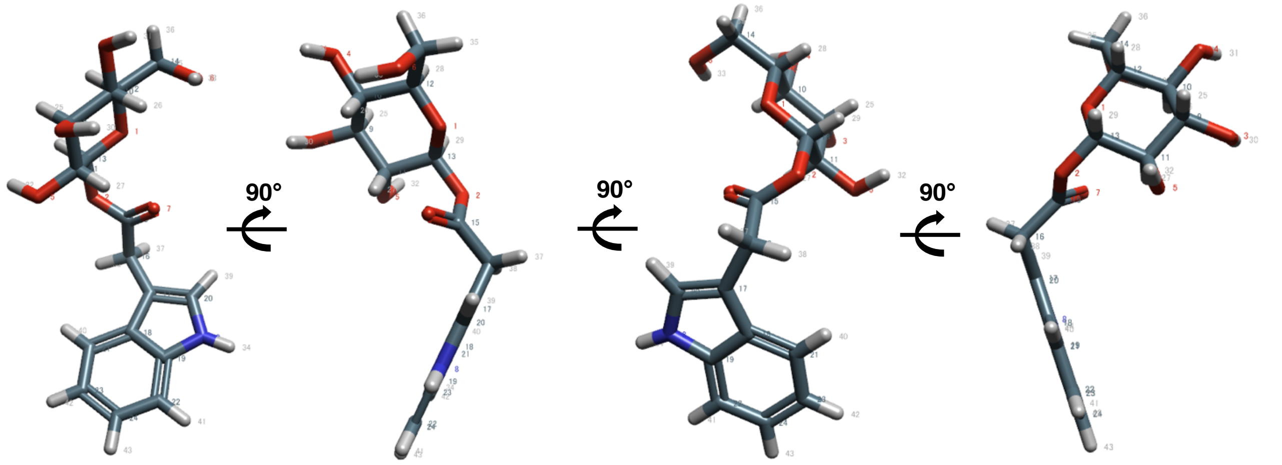

Figure S7. The atom number on the IAA-Glc that was identified in Flare software

The chemical structure of IAA-Glc is shown as a stick model with atom colors denoting oxygen (red), carbon (gray), nitrogen (blue) and hydrogen (light gray). The numbers shown on the side of each atom indicate the atom number in IAA-Glc.

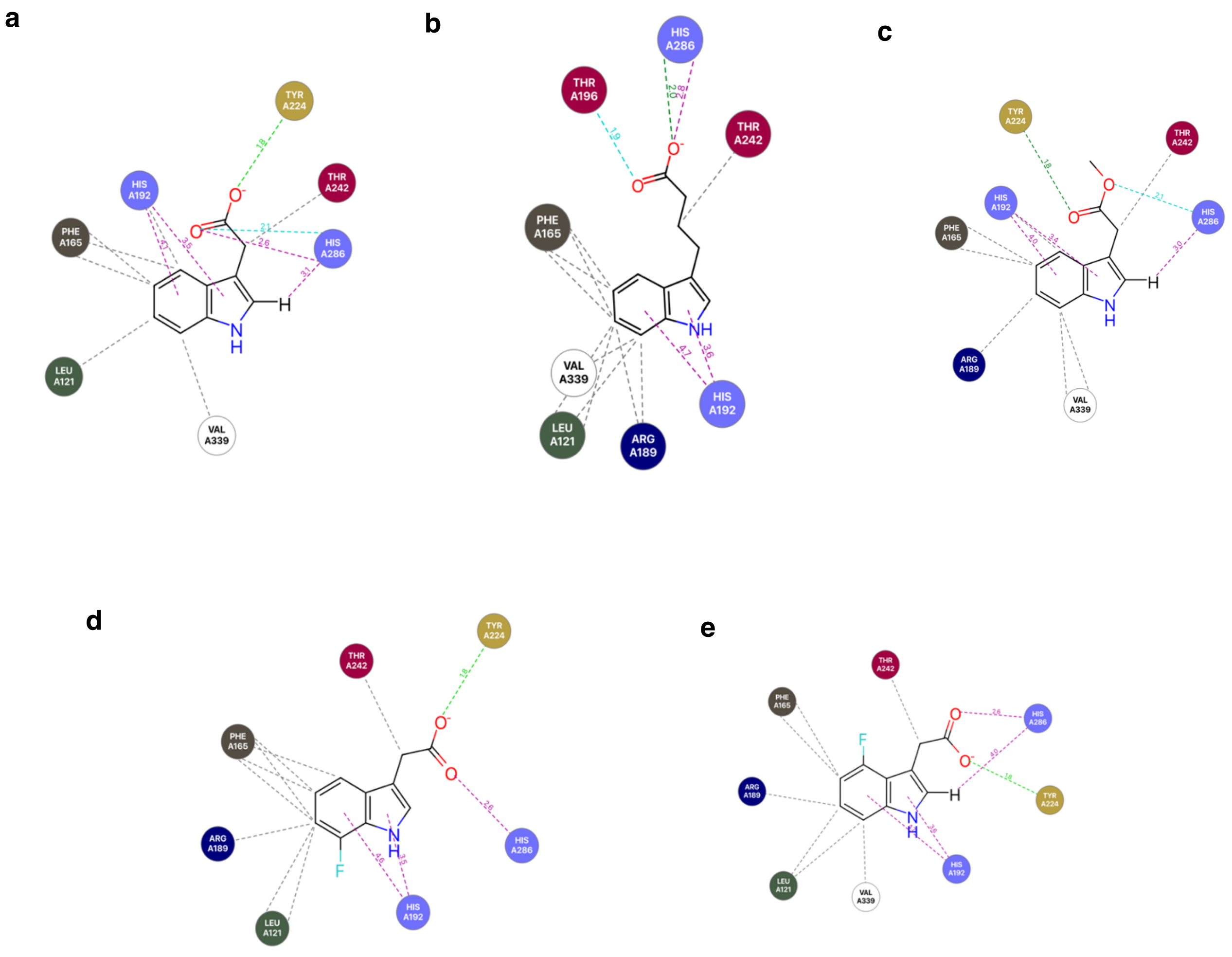

Figure S8. Simulated interaction maps of fragment-like compounds tested against TGW6

(a) IAA, (b) IBA, (c) MeIAA, (d) PK0-38511 and (e) PK0-38501. Interacting residues are represented as colored circles. The dotted lines indicate the kind of interaction and the position of interaction: hydrophobic interaction (gray), aromatic-aromatic contacts (purple), hydrogen bonds and weak hydrogen bonds (green and light blue), respectively.

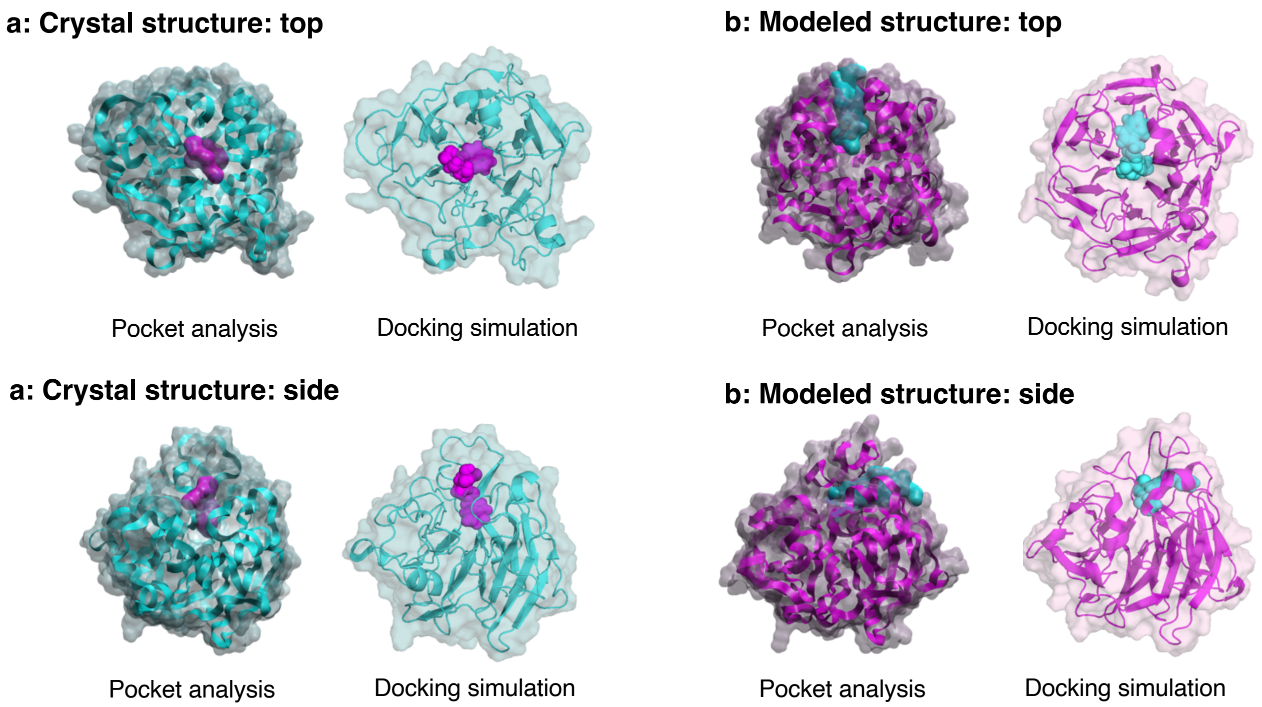

Figure S9. The detected pocket and binding position of IAA-Glc in TGW6 based on the crystal structure and the modeled structure

(a) The detected shape of the pocket (right) and the binding position of IAA-Glc (left) in the crystal structure of TGW6 (PDB: 8KG3). TGW6 is represented as a ribbon and surface model in cyan for the pocket analysis. The detected pocket is shown as a magenta object. For the docking simulation, TGW6 is shown as a cartoon and surface model in cyan. The simulated binding of IAA-Glc is represented as a spheres model in magenta. (b) The detected shape of the pocket (right) and the binding position of IAA-Glc (left) in the modeled structure of TGW6 (Ishimaru and Hirotsu *et al. Nat. Genet.* 2013). TGW6 is represented as a ribbon and surface model in magenta for the pocket analysis. The detected pocket is shown as a cyan object. For the docking simulation, TGW6 is shown as a cartoon and surface model in magenta. The simulated binding IAA-Glc is represented as a spheres model in cyan.
